# Overcoming BCR::ABL1 dependent and independent survival mechanisms in chronic myeloid leukaemia using a multi-kinase targeting approach

**DOI:** 10.1186/s12964-023-01363-2

**Published:** 2023-11-29

**Authors:** Caroline Busch, Theresa Mulholland, Michele Zagnoni, Matthew Dalby, Catherine Berry, Helen Wheadon

**Affiliations:** 1 Paul O’Gorman Leukaemia Research Centre, School of Cancer Sciences, University of Glasgow, Glasgow, G12 0ZD UK; 2 Centre for Microsystems and Photonics, Electronic and Electrical Engineering, University of Strathclyde, Glasgow, G1 1XW UK; 3 Mazumdar-Shaw Advanced Research Centre, School of Molecular Biosciences, University of Glasgow, Glasgow, G11 6EW UK

**Keywords:** Chronic myeloid leukaemia, Leukaemic stem cells, Bone morphogenetic protein, Multi-kinase drug targeting

## Abstract

**Background:**

Despite improved patient outcome using tyrosine kinase inhibitors (TKIs), chronic myeloid leukaemia (CML) patients require life-long treatment due to leukaemic stem cell (LSC) persistence. LSCs reside in the bone marrow (BM) niche, which they modify to their advantage. The BM provides oncogene-independent signals to aid LSC cell survival and quiescence. The bone-morphogenetic pathway (BMP) is one pathway identified to be highly deregulated in CML, with high levels of BMP ligands detected in the BM, accompanied by CML stem and progenitor cells overexpressing BMP type 1 receptors- activin-like kinases (ALKs), especially in TKI resistant patients. Saracatinib (SC), a SRC/ABL1 dual inhibitor, inhibits the growth of CML cells resistant to the TKI imatinib (IM). Recent studies indicate that SC is also a potent ALK inhibitor and BMP antagonist. Here we investigate the efficacy of SC in overcoming CML BCR::ABL1 dependent and independent signals mediated by the BM niche both in 2D and 3D culture.

**Methods:**

CML cells (K562 cell line and CML CD34^+^ primary cells) were treated with single or combination treatments of: IM, SC and the BMP receptors inhibitor dorsomorphin (DOR), with or without BMP4 stimulation in 2D (suspension) and 3D co-culture on HS5 stroma cell line and mesenchymal stem cells in AggreWell and microfluidic devices. Flow cytometry was performed to investigate apoptosis, cell cycle progression and proliferation, alongside colony assays following treatment. Proteins changes were validated by immunoblotting and transcriptional changes by Fluidigm multiplex qPCR.

**Results:**

By targeting the BMP pathway, using specific inhibitors against ALKs in combination with SRC and ABL TKIs, we show an increase in apoptosis, altered cell cycle regulation, fewer cell divisions, and reduced numbers of CD34^+^ cells. Impairment of long-term proliferation and differentiation potential after combinatorial treatment also occurred.

**Conclusion:**

BMP signalling pathway is important for CML cell survival. Targeting SRC, ABL and ALK kinases is more effective than ABL inhibition alone, the combination efficacy importantly being demonstrated in both 2D and 3D cell cultures highlighting the need for combinatorial therapies in contrast to standard of care single agents. Our study provides justification to target multiple kinases in CML to combat LSC persistence.

**Supplementary Information:**

The online version contains supplementary material available at 10.1186/s12964-023-01363-2.

## Abstract (plain language summary)

Blood is made in the spongy inner most section of the bone, called the bone marrow. The bone marrow is where normal blood stem cells live that are responsible for producing the different blood cell types; white blood cells (fight infections), red blood cells (carrying oxygen around the body), platelets (blood clotting) and other cells which support this process. Chronic myeloid leukaemia (CML) is a type of blood cancer that starts in the bone marrow. CML occurs when a normal blood stem cell becomes damaged, forming a leukaemia stem cell (LSC), leading to blood cancer. LSCs multiply and generate many faulty cancerous white blood cells that do not work properly. Patients are treated with a drug called imatinib, which reduces the number of cancerous cells circulating in the body. In many cases, this treatment is not enough to cure the disease because the bone marrow protects the LSCs from the drug meaning patients must remain on long term treatment. This work has discovered one of the ways in which the bone marrow protects LSCs from treatments and has used this knowledge to test new drugs that stop this protection. Our findings show that by combining two drugs, one that overcomes this protection and one that directly targets the cancerous cells, we can destroy more of the LSCs. These findings are a step closer towards a cure for CML and could improve treatment for patients in the future.

Video Abstract

**Supplementary Information:**

The online version contains supplementary material available at 10.1186/s12964-023-01363-2.

## Background

Chronic myeloid leukaemia (CML) accounts for approximately 15% of all newly diagnosed leukaemia cases. Patient treatment using tyrosine kinase inhibitors (TKIs) such as imatinib (IM) has improved the disease outcome of patients. However, patients stay on life-long treatment and more advanced-phases are less responsive to treatment and can still relapse with therapy, thus TKIs are not curative [1]. Secondary (acquired) resistance can occur in patients during TKI therapy [2, 3]; with half of the non-responders displaying point mutations within the kinase domain of *BCR::ABL1* thus blocking TKI binding [4]. Patients also fail TKI treatment without displaying these kinase mutations [5] and with no detectable *BCR::ABL1* present [6]. Past studies indicate CML leukaemic stem cell (LSC) survival is *BCR::ABL1* kinase-independent [7], and persistence of TKI-insensitive LSCs is thought to underpin our inability to cure more patients [8]. Strategies beyond targeting BCR::ABL1 are required to overcome the hurdle of LSC persistence. One possible approach is targeting alternative survival, proliferation and self-renewal mechanisms known to be deregulated in CML in combination with TKIs [9–12].

Hyperactivation of members of the Src family kinases (SFKs) in CML [13] led to the development of second generation TKIs targeting BCR::ABL1 and SFKs. SFK inhibitors have shown good efficiency in CML and other haematological disorders [14]. Another deregulated pathway in CML patients is the bone-morphogenetic pathway (BMP) with alterations most prevalent in TKI resistant patients [15–18]. In 40% of newly diagnosed patients, high levels of BMPR1B/ALK6 are detected, which can act as a driver for myeloid progenitor expansion. Moreover, a higher abundance of BMP2 and BMP4 in the BM plasma of CP-CML patients at diagnosis was determined compared to normal donors [15]. Data from our group showed that dual targeting of BMP pathway signalling and BCR::ABL1 using IM showed higher efficacy than IM alone [17]. Saracatinib (SC), a SRC/ABL1 dual inhibitor, inhibits the growth of CML cells resistant or sensitive to IM, as a consequence of down regulating important survival signalling pathways (STAT5, ERK, PI3K/AKT) [1, 19]. SC’s activity on ABL1 has been tested in CML in vivo models [20], and in Phase II trials for other cancers. Recent studies indicate that SC is a potent ALK inhibitor especially against ALK 2, 3 known to be upregulated in CML [15–18] as well as a BMP antagonist [21, 22]. SC due to its low toxicity levels, may present, in combination with other drug targets a therapeutic window in CML to overcome LSC BCR::ABL1 dependent and independent signals in the BM niche.

In this study, we evaluate SRC/ABL1/ALK inhibition against ABL1 alone and in combination with a specific ALK 2, 3 and 6 inhibition and demonstrate the advantage of targeting BCR::ABL1 independent survival mechanisms both in the presence and absence of BM protective mechanisms, to come one step closer to eliminating low-level disease persistence.

## Methods

### Patient samples

Normal and CML samples were taken at diagnosis following informed consent in accordance with the Declaration of Helsinki, and stored for future use by approval of the NHS Greater Glasgow and Clyde West of Scotland Research Ethics Committee (REC reference 15/WS/0077). Samples were processed as previously described [17]. The CD34^+^-enriched stem and progenitor population from normal and CML cells were thawed and cultured overnight in serum free medium (SFM) supplemented with; IL-3, IL-6 and G-CSF (20 ng/mL), SCF and FLT3L (100 ng/mL) (200–03, 200–06, 300–23, 300–07, 300–19, PeproTech). Prior to inhibitor treatment media was changed to physiological growth factor concentrations; IL-3, IL-6 and G-CSF (0.2 ng/mL), SCF and FLT3L (1 ng/mL).

### Cell lines

K562 cells (DSMZ) were grown as previously described [17]. HS-5 cells were grown in DMEM (21969–035, Gibco) with 10% fetal calf serum, 1 mM glutamine and 1% penicillin–streptomycin (16141–079, 25030024, 15140122, Invitrogen). Cell line authentication test was performed by Eurofins Genomics prior to carrying out these experiments, 100% alignment with DSMZ and ATAT databases. For co-culture studies, HS-5 were seeded 24 h before on collagen coated 12-well plates (M9187, Greiner Bio-One) at a cell density of 1.2 × 10^5^ cells per well.

### Inhibitors

100 mM imatinib (IM) (S2475, Selleckchem) stock in water was stored at 4˚C; Dorsomorphin (DOR) (ab120843, Abcam) and saracatinib (SC) (S1006, Selleckchem) 10 mM stocks in DMSO stored at -80˚C. Inhibitors were diluted in complete medium as required prior to cell treatments. BMP4 (AF-120-05ET, Peprotech) stock solution of 10 µg/mL in 2% BSA/PBS was stored at -80˚C. For all experiments a final concentration of 20 ng/mL was used.

### Resazurin assay

Half maximal inhibitory concentration (IC50) of SC after 24 h, 48 h and 72 h was established using resazurin assay (R7017-5G, Sigma-Aldrich) according to the manufacturer’s instructions. The IC50 was calculated using GraphPad Prism 8.

### Drug combination studies (CompuSyn)

CompuSyn software (ComboSyn, Inc.) was used to investigate the synergism of the inhibitors SC and DOR in Resazurin assays. The software was based on the Chou-Talalay method for drug combination based on the median-effect equation derived from the mass-action law principle [23, 24]. The CI provides a quantitative definition for additive effect (CI = 1), synergism (CI < 1) or antagonism (CI > 1) in drug combinations.

### MSC spheroid formation

MSC spheroids were formed in 24-well AggreWell 400 plates (34815, Stem Cell Technologies). MSCs were seeded at 1.2 × 10^5^ cells/well (100 cells/spheroid) alone, or in co-culture with leukaemic cells (0.6 × 10^5^ cells/well). 24 h after spheroid formation, mono- or co-cultured cells were treated with TKIs, BMP inhibitor or a combination for 72 h. MSC spheroids were digested into single cell suspension by collagenase D (2.5mg/mL) (11088858001, Roche).

### Flow cytometry

For apoptosis analysis 5 × 10^4^ cells were stained with Annexin V-FITC/7AAD (556419, 559925, BD Biosciences). MSC spheroid cells were stained for Annexin V-FITC and propidium iodide (PI) (P-4864, Sigma-Aldrich) after separation into single cells using collagenase D (2.5mg/mL) (11088858001, Roche). PI buffer (550825, BD Biosciences) was used as described by the manufacturer to assess cell cycle progression. For cell division analysis normal and CP-CML CD34^+^ cells were labelled with CellTrace™ Violet (CTV) (C34557, Thermo Fisher) as described by manufacturer. For establishing a maximum point of fluorescence staining, cells were cultured with Colcemid (100 ng/mL, 10295892001, Roche) to determine non-dividing cells (CTV max), in addition cells were labelled for CD34^+^ (555824, BD Biosciences). To distinguish cell types in co-cultures, MSCs were labelled with anti-CD73-PE (550257, BD Biosciences) and K562 cells with anti-CD235A-APC (551336, BD Biosciences) prior to apoptosis staining. Data analysis was performed by Flow-Jo-V10 software.

### Microfluidic devices

Microfluidic devices were provided by the Zagnoni group and fabricated as described previously [25]. Devices comprised of seven channels with two open wells. Each channel had three grids and each grid had forty-two microwells. MSC cell suspension was prepared ranging between 6–8 × 10^6^ cells/ml. 5–8 µL of cell suspension was injected in one of the wells, allowing cells to accumulate in the microwells. For co-culture experiments a cell solution of the same concentration and a cell ratio of 3:1 between MSCs and K562 was used.

### Viability staining and co-localization studies

After 72 h cells were stained for viability with 8 µg/mL fluorescein diacetate (FDA) (F7378-5G, Sigma Aldrich), 20 µg/mL PI (81845-25MG. Sigma Aldrich), and 5 µM Hoechst33324 (62249, Thermo Fisher). Microfluidic devices were incubated for 20 min and dye was exchanged after 10 min. Excess dye was removed with two washing steps of PBS for 5 min. Devices were imaged immediately after staining (Observer A1, Zeiss connected to an Orca Flash 4.0 camera Hamamatsu). Fluorescent images were processed in MATLAB software to assess spheroid size before and after inhibitor treatment as well as the percentage of viable cells. To study co-localization of leukaemic cells pre-labelled with CTV and live/dead stains, a co-localization plug-in called JACoP [26] was run in ImageJ. Within the plug-in, Costes automatic threshold was applied. The resulting M2 coefficient (Live/Dead fraction overlapping with CTV fraction) for each PI and FDA stain were added up and set to 100% to individually generate the percentage of live and dead stained cells, which are also CTV positive. Afterwards, all values were normalized to NDC and M2 values plotted using GraphPad Prism 8 and investigated for differences between treatments running a One-way ANOVA.

### Protein analysis

Cell lysates were prepared and 30 µg of protein resolved by western blotting as described previously [27]. The antibodies used are provided in Suppl. Table 1. Primary antibodies were detected with secondary fluorescent antibodies (926–68072, IRDye 680RD donkey anti-mouse or 926–32213, IRDye 800CW donkey anti-rabbit, LICOR). Signal intensities were quantified by densitometry using ImageStudioLight (LICOR) and statistically analysed using a One-Way-ANOVA, GraphPad Prism 8.

### Quantitative real-time polymerase chain reaction

Quantitative real-time polymerase chain reaction (qRT-PCR) was run using the Fluidigm BioMark HD System platform (Applied Bio-systems) as described per manufacturer’s instructions. Eight low/medium copy number reference genes were used with a list of primers provided in Suppl. Table 2, reference genes shaded in grey.

### Colony forming cell assay (CFC)

Following 72 h treatment 4,000 cells were inoculated into 3 mL of a semisolid medium (MethoCult H4034, Stem Cell Technology). Cells were cultured for 10–14 days, and colonies counted and scored into 5 categories: BFU-E (burst-forming unit-erythroid), CFU-G (colony-forming unit- granulocyte), CFU-M (colony-forming unit- macrophage), CFU-GM (colony-forming unit- granulocyte, macrophage), CFU-GEMM (colony-forming unit- granulocyte, erythroid, macrophage, megakaryocyte) using an inverted EVOS microscope.

### Gene set enrichment analysis (GSEA)

GSEA [28, 29] was performed using the GSEA software v4.2.2 to determine whether genes are differentially expressed in CP-CML samples treated with SC + DOR compared to NDC. Reference gene sets from the Molecular Signatures Database (MSigDB) v7.5.1 of c5 (c5.bp.v5.2.symbols.gmt). The MSigDB c5 is a pathway gene set annotated by the same GO terms [30]. The number of permutations was set at 1,000. Enrichment results satisfying a nominal *P*-value cut-off of < 0.05 with a false discovery rate (FDR) > 0.25 were considered statistically significant.

### Statistics

All data are presented as mean ± standard deviation (SD) unless otherwise stated. All statistical analyses were performed using GraphPad Prism 8. When comparing between-groups one-way ANOVAs and when comparing multiple groups (untreated and treated samples of CML LSCs and normal HSCs) two-way ANOVAs were performed. *P*-values of < 0.05 were considered as significant. Significance is indicated by asterisks (**** < 0.0001, *** 0.0001 to 0.001, ** 0.001 to 0.01, * 0.01 to 0.05).

## Results

### Dual targeting of SRC and ABL1 with saracatinib

**Fig. 1:**
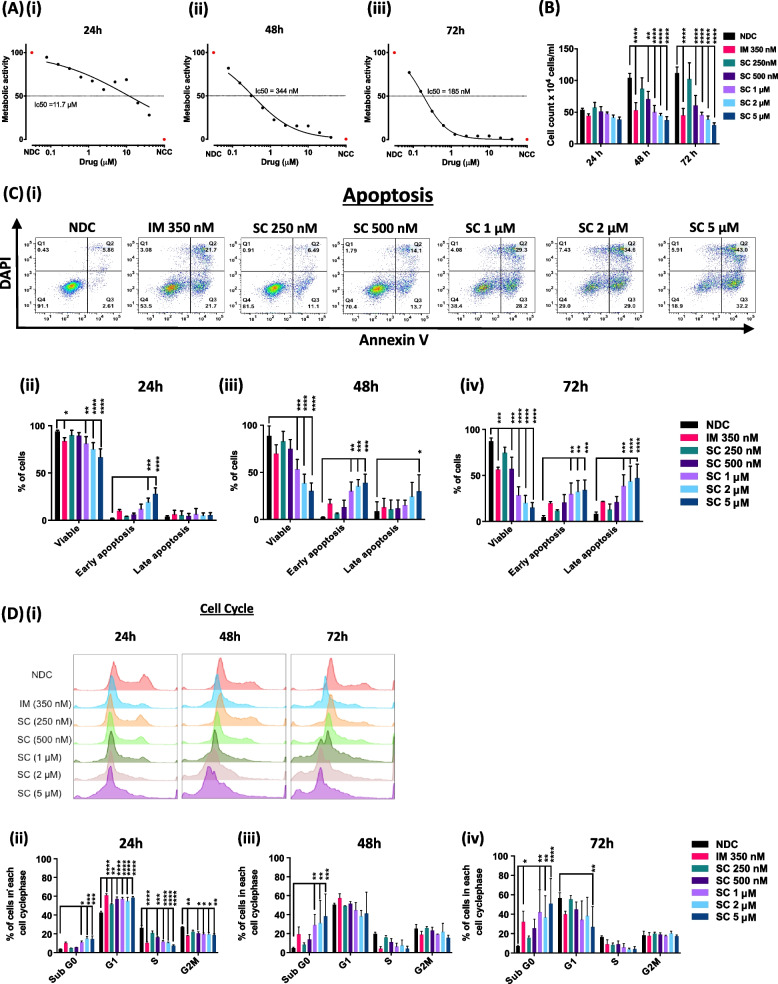
Effects of SC on CML cell line K562. **Ai-iii** K562 cells were treated with different SC concentrations and metabolic activity monitored over 72 h to establish an IC_50_. **B** Cells were treated with SC (250 nM, 500 nM, 1 µM, 2 µM or 5 µM) or IM (350 nM) over 72 h and viable cells counts determined by trypan blue exclusion. **Ci-iii** Apoptosis was measured by flow cytometry. Example gates taken from FlowJo V10 showing one measured set of the three replicate experiments at 72 h. **Di-iii** K562 cells were fixed after 24 h, 48 h and 72 h followed by DAPI staining to determine the percentage of cells in each stage of cell cycle. The histograms demonstrate the cell cycle distribution of each treatment arm for 24 h, 48 h and 72 h for one replicate. All data are expressed as mean ± SD (*n* = 3) and treatments were compared to the NDC by Two-Way-ANOVAs in GraphPad Prism 8. The grade of significance is indicated by asterisks (**** *p* < 0.0001, *** *p* 0.001 to 0.0001, ** *p* 0.01 to 0.001, * *p* 0.05 to 0.01)

To determine how effective the SRC/ABL1 inhibitor SC was at preventing cell proliferation and survival, we identified a working range for the cell line K562. The half-maximal inhibitory concentration (IC50) was determined by resazurin assay and trypan-blue exclusion assays after 24, 48 or 72 h to define an inhibitor dose range for subsequent apoptosis and cell cycle assays, with IM (IC50) used as a comparator for SC efficacy (Fig. 1A & B). Apoptosis analysis revealed a significant increase in apoptotic cells with increasing SC concentrations (Fig. 1C). The outcome of the apoptosis data was also reflected in the cell cycle data (Fig. 1D) with cells accumulating in G1 at 24h following SC treatment and viable cell numbers decreasing, as shown by a higher percentage of cells accumulating in the Sub G0 fraction by 48 and 72h especially with higher doses of SC (1, 2 and 5µM).

**Fig. 2:**
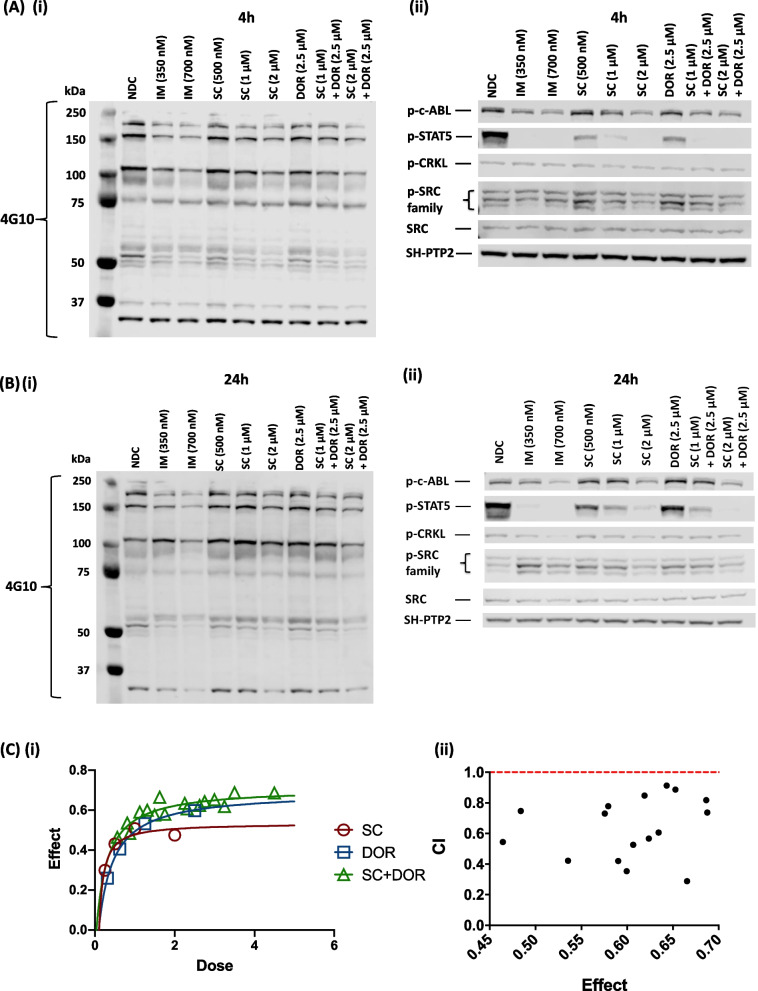
Immunoblot analysis of downstream phosphoproteins inhibited by IM, SC and DOR treatment. **Ai **+** ii** & **Bi **+** ii** K562 cells were treated with IM (0.35 µM, 0.7 µM), SC (0.5 µM, 1 µM, 2 µM) and the combination SC (1 µM or 2 µM) + DOR (2.5 µM) for 4 h or 24 h followed by analysis of down-stream targets of BCR::ABL1 signalling; p–c-Abl, p-STAT5, p-CRKL and SFKs (*n* = 3). SH-PTP2 and total SRC were used as loading control. **Ci **+** ii** CompuSyn analysis of treated CP-CML CD34.^+^ cells confirmed SC in combination with DOR works in a synergistic manner. **C-i** CompuSyn is calculating the fraction affected (Fa) of each drug on its own or combination and **C-ii** ranks those values with a combination index (CI). The CI for all drug combinations is below 1, which defines synergy. See also Figure S1

Next, we confirmed the specificity of action of SC alone and in combination with BMP pathway receptor (ACVR1/ALK2, BMPR1A/ALK3, BMPR1B/ALK6) inhibitor dorsomorphin (DOR) by immunoblotting analyses after 4 and 24 h of treatment. Overall tyrosine phosphorylation of cellular proteins was investigated (4G10), showing a decrease in phosphorylation with increasing IM and SC concentrations as well as dual treatment (SC + DOR) (Fig. 2A-i). Phosphorylation of BCR::ABL1 and its down-stream targets CRKL and STAT5 were investigated, as well as phosphorylation of SRC (Fig. 2A-ii and B-ii). P–c-ABL1 levels rapidly decreased after 4 h treatment with IM or SC, indicating both inhibitors are effective in inhibiting ABL activation (Fig. 2A-ii; Suppl. Figure 1A-i). IM (700 nM; *p* = 0.088) treated cells displayed a downregulation of c-ABL phosphorylation in a similar manner for both time points (Suppl. Figure 1A-i & B-i). p-STAT5 was significantly down regulated for all treatments at both time points (Fig. 2A-ii and B-ii + Suppl. Figure 1A-ii & B-ii). After 24 h treatment, DOR displayed the smaller reduction, whereas after 4 h it resulted in p-STAT5 downregulation in the same manner as SC (500 nM). A second down-stream target, p-CRKL, revealed a significant decrease for both dual treatments (SC 1 µM + DOR and SC 2 µM + DOR) after 4 h (Fig. 2A-ii+ Suppl. Figure 1A-iii). Dual treatments likewise caused down regulation after 24 h (Fig. 2B-ii + Suppl. Figure 1B-iii); however, only IM (700 nM) revealed a significant decrease*.* SFK members, showed a significant downregulation following SC treatment (2 µM, 4 h) (Fig. 2A-ii + Suppl. Figure 1A-iv), incubation with the 24 h time point revealed an overall up-regulation (Fig. 2B-ii + Suppl. Figure 1B-iv), thus counteracting the inhibition. These findings confirm that IM and SC are effective at targeting downstream kinases and signalling proteins activated by BCR::ABL1.

### Combinatorial targeting of SRC/ABL and the BMP pathway results in an increase in apoptosis and reduction in cell cycle progression

**Fig. 3:**
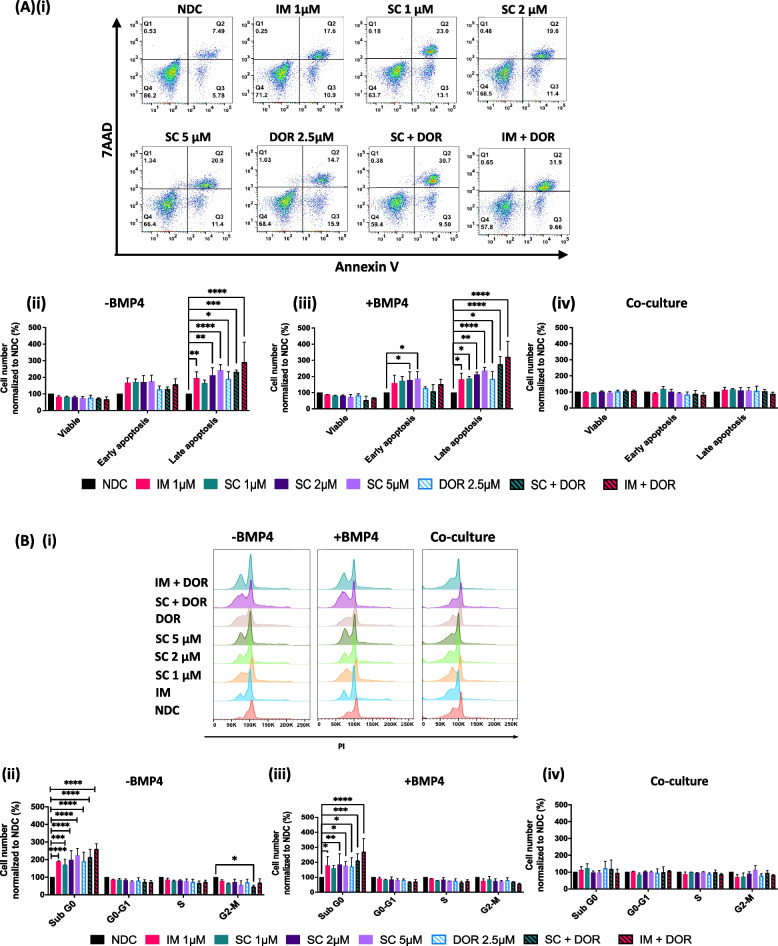
SC and DOR induce apoptosis and an increase in Sub G0 in CP-CML CD34^+^ cells. **A** Annexin V/ 7AAD apoptosis analysis of CP-CML CD34^+^ cells treated with the TKIs IM or SC and BMP receptor inhibitor DOR and the combination (IM = 1 µM, SC = 1 µM, 2 µM, 5 µM; DOR = 2.5µM; Combo = 1 µM TKI (SC or IM) + 2.5 µM DOR) in absence or presence of BMP4 (20 ng/ml) or in co-culture with HS-5 at 72 h. **Aii **+** iii** Apoptosis increases in CP-CML CD34.^+^ samples after treatment with SC and DOR in the presence and absence of BMP4. **Aii-iv** Comparison of mono- and co-culture conditions reveal an increase in viable cells and a decrease of cells in late apoptosis when co-cultured with HS-5. **Bi + ii** Cell cycle analyses shows a significant increase in the number of cells in Sub-G0 in all single and dual treatments of CP-CML samples. **Biii** Similar results were observed in presence of BMP4. **Biv** HS-5 co-cultures showed an overall protective effect with no changes observed after treatment. Data are expressed as mean ± SD (*n* = 3) and were compared using Two-Way-ANOVA (**** *p* < 0.0001, *** *p* 0.001 to 0.0001, ** *p* 0.01 to 0.001, * *p* 0.05 to 0.01). See also Figure S2

After confirming the efficacy of SC in the CML cell line K562 we investigated the effect on primary CP-CML CD34^+^ cells compared to normal CD34^+^ cells. After 72 h treatment, we performed apoptosis and cell cycle analysis. To mimic BMP levels in the BM of patients we supplemented some cultures with the ligand BMP4 (20 ng/mL) and studied co-culture behaviour with the stromal cell line HS-5. Inhibition of BCR::ABL1 through SC or IM and ALKs through DOR shifted the CML cells into apoptosis, reducing the number of viable cells (Fig. 3A-i-ii). Results were more profound in the CP-CML CD34^+^ cells when TKIs were used in combination with DOR, with significantly higher levels of apoptosis observed in the CP-CML CD34^+^ (Fig. 3A-i-ii); the synergistic effect was not observed with normal CD34^+^ cells (Suppl. Figure 2A-i-ii). Similar results were observed in the presence of BMP4 (Fig. 3A-iii). Co-cultures on HS-5 protected CP-CML CD34^+^ and normal CD34^+^ cells from undergoing apoptosis in response to TKIs and BMP pathway inhibition (Fig. 3A-iv). Cell cycle analysis at 72 h (Fig. 3Bi-iii) revealed CP-CML CD34^+^ cells were affected by inhibition with DOR alone or in combination with a TKI. Normal CD34^+^ cells were also affected albeit to a lesser effect (Suppl. Figure 2B-i-iii). In line with our apoptosis data, CP-CML CD34^+^ cells in the Sub G0 stage increased proportionally to the dose of SC and even further in the combination arms (Fig. 3B-ii). Similar results were observed in BMP4 supplemented samples (Fig. 3B-iii) with the only non-significant effect seen with IM. Also, in line with the apoptosis data, HS-5 co-cultured samples did not display any difference compared to the NDC (Fig. 3B-iv). These findings suggest a protective effect is being conferred to the leukaemic cells by the stroma to overcome the inhibitory effects of the drug treatments.

### Dual targeting of CP-CML CD34^+^ cells with TKI and ALKs inhibitor affects cell proliferation and differentiation potential and reduces CD34^+^ cell numbers

**Fig. 4:**
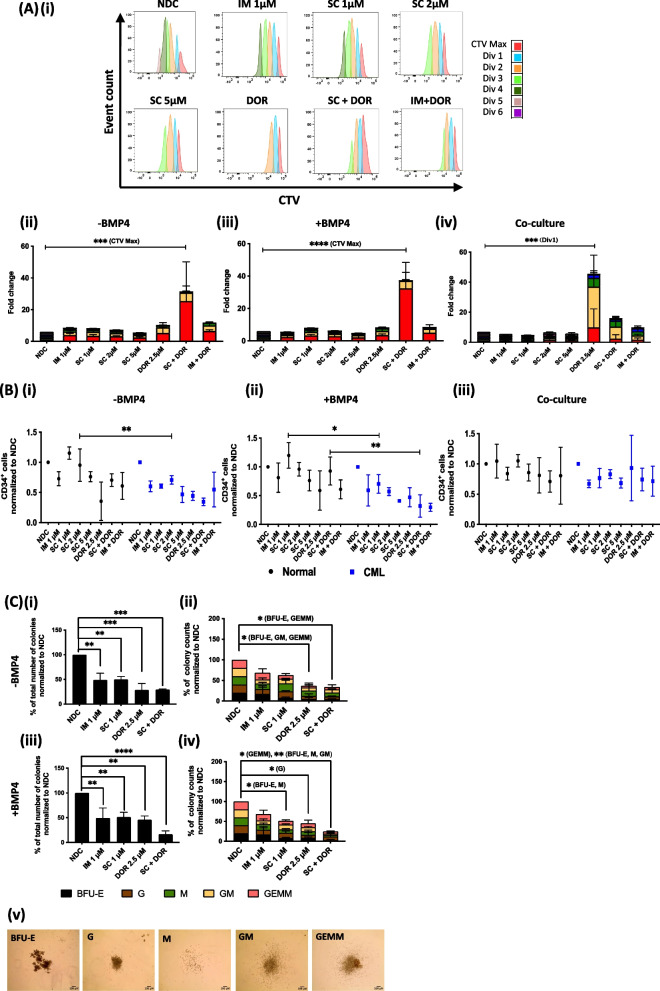
Dual targeting of CP-CML CD34^+^ cells reduces cell proliferation, CD34^+^ cell and colony numbers. **Ai-iv** CTV proliferation analysis of CP-CML cells treated with IM,SC or DOR and the combination (IM = 1 µM, SC = 1 µM, 2 µM, 5 µM; DOR = 2.5µM; Combo = 1 µM TKI (IM or SC) + 2.5 µM DOR) in absence or presence of BMP4 (20 ng/ml) or in co-culture with HS-5 at 72 h. **Aii-iv** Proliferation analysis of CP-CML samples indicates that DOR in combination with TKIs synergistically inhibits proliferation compared to single treatment. SC + DOR alone display the biggest fold change compared to NDC in all culture conditions. **Bi-iii **TKIs and DOR reduce CD34^+^ cell numbers in CP-CML samples and to a lower extent in normal samples. **Bi** Biggest reduction was observed with SC 5 µM, DOR and combinatorial treatments. **Bii** Similar results were obtained in presence of BMP4. **Biii** Co-cultures displayed a decrease for all treatments but to a lower extent compared to monocultures. Microscopic analysis revealed a reduction in **Ci **+** iii** total colony counts and (**Cii **+** iv**) types but no visible change in (**Cv**) colony morphology. TKI and DOR alone and in combination reduced (**Ci **+ **ii**) total colony counts and affected different (**Cii **+** iv**) colony types independent of BMP4 presence in CP-CML samples. Data are expressed as mean ± SD (*n* = 3). CTV_max_ and division 1 were compared using Two-Way-ANOVA, normalized CD34.^+^ values and colony counts were compared using Two-Way-ANOVA (**** *p* < 0.0001, *** *p* 0.001 to 0.0001, ** *p* 0.01 to 0.001, * *p* 0.05 to 0.01). See also Figure S3 and S4

Next, we evaluated proliferation/cell division and total CD34^+^ cell numbers. Both CML CD34^+^ cells (Fig. 4A-i-iv) as well as normal CD34^+^ cells (Figure Suppl. 3A-i-iv) were sensitive to BMP pathway inhibition and underwent less cell divisions when treated compared to the NDC. CP-CML CD34^+^ cells showed enhanced sensitivity in all treatment arms, with more cells accumulating in CTVmax and early divisions, with the most dramatic effect observed with SC + DOR (Fig. 4A-i-iv). A similar effect was seen in the presence of BMP4 (Fig. 4A-iii). Co-culture on HS-5 diminished the efficiency of the drugs, however CML cells still accumulated in CTVmax and division 1 following DOR, SC + DOR and IM + DOR treatment, indicating the BMP inhibitor was causing an effect, albeit to a reduced capacity (Fig. 4A-iv). TKIs and DOR lead to a reduction in the number of CD34^+^ cells in CP-CML samples compared to normal samples (Fig. 4B-i + ii), with a significant reduction observed following SC (2 µM) treatment without BMP4 (supplement (Fig. 4B-i)), and SC (1 µM) and the combination of SC + DOR with BMP4 (supplement (Fig. 4B-ii)). All treatment arms led to an overall reduction of CD34^+^ cells. However, after 72 h with inhibitors normal samples expressed double the number of CD34^+^ cells compared to CML samples. CD34^+^ cell numbers in HS-5 co-cultured samples stayed relatively high in total after 72 h of treatment but displayed a notable reduction with TKIs (Fig. 4B-iii). To investigate differentiation and proliferation potential following treatment CFC assays were performed. In CP-CML samples, all treatment arms led to a significant decrease in total colonies independent of BMP4 presence (Fig. 4C-i + ii). Different types of colonies were also affected, such as BFU-E and GEMM colonies under DOR and combination treatment (SC + DOR) in the absence of BMP4 (Fig. 4C-ii). Further, in the presence of BMP4 (Fig. 4C-iv), M and GM colonies were additionally reduced in numbers. DOR treatment also led to a reduction in total colony counts in normal samples (supplement (Fig. 4B-i-iv)). The CTV and CFC findings indicate that targeting multiple kinases reduces the survival of CML stem/progenitor cells.

### Combinatorial treatment with TKI and BMP pathway inhibitor affects expression of genes involved in apoptosis, proliferation, cell cycle and Wnt signalling

**Fig. 5:**
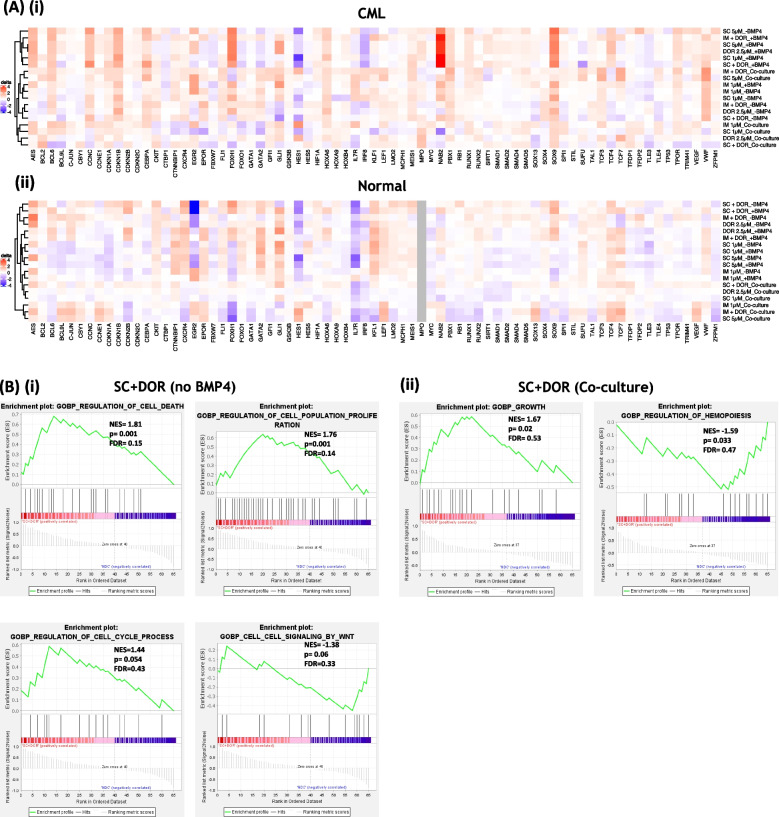
Gene expression comparison analyses of CP-CML and normal CD34^+^ cells following drug treatments. **Ai + ii** Fluidigm gene expression analyses of CP-CML and normal CD34^+^ cells treated with IM, SC or DOR, and the combination (IM = 1 µM, SC = 1 µM, 2 µM, 5 µM; DOR = 2.5 µM; Combo = 1 µM TKI (IM or SC) + 2.5 µM DOR) in absence or presence of BMP4 (20 ng/ml), or in co-culture with HS-5 at 72 h. Gene expression heat maps (-ddCt) of treated CP-CML and normal CD34.^+^ cells resulted in treatment clustering for culture conditions. **B** GSEA enrichment plots for CP-CML (**i**) monocultures without BMP4 and (**ii**) co-culture treated with SC + DOR shown together with nominal enrichment score (NES), p-value and False Discovery Rate (FDR). Positive values indicate upregulation of gene sets and negative values indicate downregulation of gene sets. See also Figure S5 and S6

Next, we investigated the effects of SC and DOR on normal and CP-CML patient CD34^+^ cells, in the different culture conditions on gene expression levels. We focused on highly interacting networks involved in; apoptosis, cell cycle regulation, proliferation, early differentiation, self-renewal and morphogenetic pathways (Supplem. Figure 5B). The different culture conditions (Supplem. Figure 5A) highlighted distinct variations in gene expression patterns. In CP-CML cells (Supplem. Figure 5A-i) we observed a downregulation of *VWF* and *VEGF* in cultures supplemented with BMP4, but an upregulation in HS-5 co-culture. Conversely, *NAB2, IL7R, CDKN1A, BCL2* and *BCL6* were upregulated with BMP4 supplementation and downregulated in co-culture conditions. Similarly, in normal control samples (Supplem. Figure 5A-ii) we noted a downregulation of the genes *VWF, PBX1, MEIS1 GSK3B, GFI1, CTBP1* and *CCNE1* in two out of three BMP4 supplemented monocultures, which were upregulated in co-cultures. By contrast, *ZFPM1, VEGF, TPOR, TAL1,* and *SMAD1* were upregulated in BMP4 supplemented monocultures and downregulated in HS-5 co-cultures (Supplem. Figure 5A-ii). Treatments were clustered for all genes in averaged CP-CML (Fig. 5A-i) and normal CD34^+^ samples (Fig. 5A-ii). Sample clustering revealed a clear division in normal samples (Fig. 5A-ii, in which samples tended to cluster for co-culture, TKI treatment, and BMP inhibitor treatment. In CML samples (Fig. 5A-i) only 5 out of 6 co-culture conditions were clustered together, with DOR being interspersed with TKI treatments. Gene enrichment analyses (GSEA) of CP-CML samples treated with SC + DOR confirmed differential expression in pathways involved in cell death, proliferation, cell cycle and Wnt signalling. For monocultures treated with the combination we observed an upregulation in cell cycle genes (*CCNC*, *CDKN1A*, *CDKN1B*; Figure S6A). These data may indicate a block in progression from G1 to S phase in CP-CML CD34^+^ cells, in line with the increase in *CCNC* expression, which does not occur in normal CD34^+^ cells and allows cells to maintain normal cell cycle. GSEA analyses along with our flow cytometry data displayed an upregulation in cell death together with an upregulation of pro-survival genes (*BCL2*, *BCL6*). Our GSEA analyses revealed an upregulation in proliferation and a downregulation in Wnt signalling. Along with those results, we identified *SOX9*, *VEGF*, *AES* and *TCF4* being particularly deregulated upon treatment and culture condition. SOX9 cooperates with canonical Wnt signalling, another morphogenetic pathway tightly connected with BMP signalling, to drive cancer progression [31]. Treatments stimulated with BMP4 showed an increase in expression for *TCF4* and *AES*, with the later acting as transcriptional repressor in the absence of β-catenin (Figure S6E). The enhanced expression of *AES* after BMP4 stimulation, has also been seen when comparing non-treated samples suggesting that BMP4 is indirectly or directly stimulating the Wnt pathway. Treatments with our inhibitors seemed to further increase levels of *AES*. In addition to GSEA enriched pathways we also found genes involved in self-renewal processes differentially expressed after treatment; *CKIT*, *PBX1*, *HOXA6*, *MEIS1* and *FOXH1* (Figure S6E). It is feasible that the differential expression upon treatments could be the result of selection for very primitive cells with self-renewal capacities.

### Inhibitors target leukaemia cells in more realistic 3D co-culture systems

**Fig. 6:**
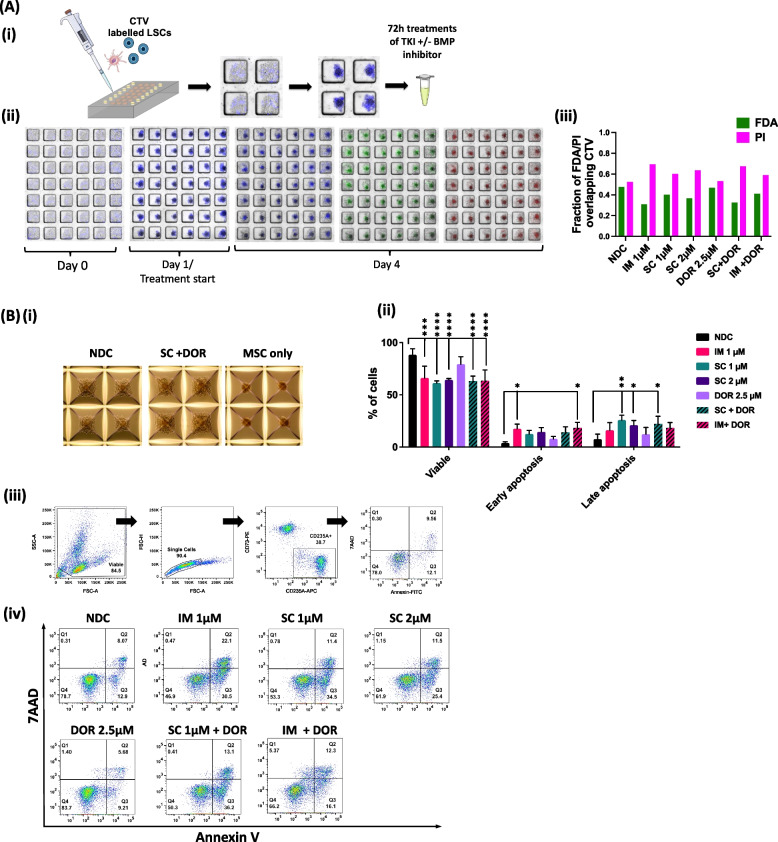
Viability studies of K562 cells co-cultured with MSC spheroids and treated with TKI ± BMP inhibitor. **Ai** Workflow schematic of pre-labelling K562 cells with the proliferation dye CTV prior to simultaneous seeding with MSCs. **Aii** Example microwells seeded with MSCs + K562-CTV, Day1- treatment start with the TKIs; IM (1 µM) or SC (1 µM, 2 µM), DOR (2.5 µM) and the combination TKI (1 µM) + DOR, and Day 4, viability staining with FDA and PI 72 h post treatment start. Blue = CTV, Green = FDA, Red = PI. **Aiii** Co-localization analyses of CTV positive and FDA/PI positive cell population were performed with the co-localization plug-in tool JACoP. Two-Way ANOVAs were performed using GraphPad Prism 8. **Bi** Co-culture studies in micropatterned plates were imaged after 72 h of drug treatment prior to subsequent CD235A-APC and CD73-PE and (**Bii-iv**) apoptosis staining. Apoptosis stains were measured by flow cytometry, expressed as mean ± SD (*n* = 3) and were compared to the NDC by Two-Way-ANOVAs in GraphPad Prism 8. The grade of significance is indicated by asterisks (**** *p* < 0.0001, *** *p* 0.0001 to 0.001, ** *p* 0.001 to 0.01, * *p* 0.01 to 0.05). See also Figure S7

As stroma-leukaemic cell interaction is crucial to evade and resist treatment in patients, we developed an in vitro system forming primary human mesenchymal stem cell (MSC) spheroids and performed co-culture experiments with the cell line K562. We used two different approaches, (i) co-culture in microfluidics with viability stain quantification (Fig. 6A) and (ii) co-culture in AggreWell plates with flow cytometry measurements (Fig. 6B). To determine how co-cultured K652 cells react to inhibitor treatments, cells were labelled for tracking with the cell proliferation dye CTV prior to simultaneous co-seeding with MSCs in the microfluidic devices. Following treatment, cells were stained in the devices for viability with FDA and PI. Co-localization analyses [26] showed that for the NDC similar amounts of viable (FDA positive) and dead (PI positive) cells (Fig. 6A-iii). For all treated samples, the percentage of PI positive/dead cells exceeding the FDA positive/viable fraction with the highest cell death observed with IM 1 µM (0.69) and SC 1 µM + DOR (0.67). Viability results obtained from microfluidic devices were compared with results obtained from AggreWell-cultured cells. After 72 h treatment spheroids were imaged (Fig. 6B-i), recovered from the microwells, and digested into single cell suspension using collagenase (2.5 mg/mL). Cells were stained for apoptosis with PI and Annexin-V-FITC followed by flow cytometric analysis (Fig. 6B-ii-iii). Except for DOR alone, all treatment arms showed an effect on viability (Fig. 6B-ii). Early apoptosis showed significant changes for IM (1 µM), SC (2 µM) + DOR (2.5 µM) and IM (1 µM) + DOR (2.5 µM). Late apoptosis revealed prominent changes only for SC (1µM) and SC (1 µM) + DOR (2.5 µM). The effect of TKIs, (IM and SC) and ALK inhibitor DOR alone or in combination on MSC spheroid viability and growth was also assessed in microfluidic devices (Suppl. Figure 6A-i-iii) and micropatterned plates (Suppl. Fig. 6B A-i-iii). Our results did not reveal any effect on cell viability (Suppl. Figure 6A-ii; B-ii) thus confirming leukaemic cell specificity of our inhibitors.

## Discussion

CML is a well-characterised and understood stem cell disease, with direct targeting of the oncoprotein BCR::ABL1 with TKI making the disease manageable for the majority of patients [32, 33]. Several clinical trials (TWISTER, STIM1, EURO-SKI, DASfree, ENESTop, LAST, DESTINY, ISAV) have shown that TKI treatment can be safely discontinued in some CP-CML patients [34–41]. Criteria guidance require that patients are on treatment for a minimum of 3 years and successfully reach a sustained deep molecular response (DMR) for at least 2 years [1]. Data from these studies indicate that successful treatment free remission (TFR) of patients with no molecular relapse at 2 years ranges from approximately 40–50%. These studies have identified key factors which influence TFR, these include; first line therapy, treatment duration, age of patient, molecular response and duration (MR4 and MR4.5), undetectable *BCR::ABL1* by digital PCR at time of discontinuation and the transcript type (Reviewed in [42]). One of the new goals in the treatment of CML patients is to ensure that more patients quickly achieve a sustained DMR so that they will be eligible to attempt TFR in the future.

Persistence of LSCs still poses a considerable hurdle in the complete eradication of the leukaemic clone and therefore TFR. In comparison to CML progenitors, which are under the influence of BCR::ABL1, LSCs have lower expression of the oncogene and therefore rely on other BCR::ABL1-independent mechanisms for their maintenance and survival. Some of the observed relapses following discontinuation of treatment may be a result of CML LSC activity, which would reconstitute BCR::ABL1 transcript levels in patients previously observed to be in DMR or having a sustained undetectable molecular response whilst maintaining a low mutational burden [6, 7, 9, 10]. This has necessitated understanding the BCR::ABL1 independent mechanisms underlying LSC persistence to devise better strategies for targeting.Targeting pathways important for survival, proliferation, self-renewal and maintenance of TKI-insensitive LSCs comes to the fore [9–12]. In CML, the BMP pathway has been revealed to be impaired in different disease stages, with almost half of newly diagnosed patients displaying high levels of BMPR1B/ALK6 [15–17, 43]. As both BMP and BCR::ABL1 signalling can activate pro-survival mechanisms there has been increasing interest in dual inhibition [17]. The TKI SC has previously shown promise in targeting IM sensitive and insensitive CML cells [19] and is currently in phase II clinical trials for other conditions. The rationale for choosing SC was its proven efficacy against ALKs and its potent BMP antagonist properties [21, 22]. In addition SC targets SFK as well as BCR::ABL1. It is known that SFK; Hck, Lyn, and Fyn strongly phosphorylate the SH3-SH2 region of BCR::ABL1 and activate aberrant signalling through STAT5 and AKT, contributing to leukaemogenesis [44, 45].

Here we demonstrate a synergistic mode of action of SC in combination with DOR, SC successfully decreased p–c-ABL levels, as well as BCR::ABL1 target p-STAT5 in the same way as IM, showing the effectiveness of SC as a BCR::ABL1 inhibitor. The combination of SC + DOR also showed a faster mode of action compared to IM, resulting in a significant reduction of the BCR::ABL1 substrate CRKL after 4 h while IM only displayed a significant reduction after 24 h. Treatment was also effective against CP-CML CD34^+^ cells. SC in combination with DOR synergistically decreased cell viability and induced apoptosis. GSEA results and expression changes of genes involved in apoptosis correlated with our flow cytometry data, with upregulation of key survival factors [46, 47] *BCL6* and *BCL2* observed upon treatment. Proliferation analyses showed an arrest in early cell divisions for the combination treatment SC + DOR. Further, inhibition of CP-CML CD34^+^ cells showed an upregulation of self-renewal gene (*CKIT*, *PBX1*, *HOXA6, FOXH1, MEIS1).* This upregulation could be the result of targeting progenitor cells [48] and the consequent selection for more primitive cells that entered a quiescent state to prevent apoptosis [7, 49]. Additionally, total colony numbers decreased after 72 h treatment with our inhibitor combination, showing that the long-term potential to proliferate and differentiate is impaired after treatment.

To mimic the high abundance of BMP4 observed in patient plasma [15] BMP4 was added to the cultures with similar findings observed following SC and DOR treatment, indicating DOR is still effective in the presence of BMP4. However, gene expression changes were observed with BMP4 supplementation. Differentially expressed genes (*AES*, *TCF4)* involved in Wnt signalling, which cross-talk with BMP signalling during development and disease were observed [50–52]. Overexpression of TCF4 or activation of Wnt signalling can stimulate the BMP2 transcript [53]. In addition, direct binding of SMAD1 and SMAD5 to AES is another way co-regulation between Wnt and BMP pathway occurs [50]. The effect of BMP ligand supplementation on other morphogenetic pathways would be interesting to assess in future work. To mimic the BM microenvironment we co-cultured CP-CML CD34^+^ cells with the stroma cell line HS-5 to investigate the stroma-leukaemia cell interaction and measure the effectiveness of our inhibitors. Hereby, we observed an overall protective effect of CP-CML CD34^+^ cells co-cultured with the stroma monolayer. This was in line with work by Garrido and colleagues who showed a protection of AML cells cultured with inhibitor when in direct contact with HS-5 cells [54]. Leukaemic cells in direct contact with stroma cells are less cycling and can go into a more quiescent state leading to chemoprotection rather than chemoresistance [55, 56]. TKIs directly target cycling cells, making them inefficient in targeting non-cycling, quiescent cells [57]. Having fewer cycling cells is supported by our proliferation data. There we observed that DOR and SC + DOR treatment resulted in an accumulation in early cell divisions. That could be the result of treatment pressure, which might be higher when targeting the BMP pathway, forcing the cells into a more quiescent state. Gene expression data also showed the protective function of the stroma layer, with no upregulation of cell cycle inhibitor genes such as *CDKN1A, CDKN2B*, involved in cell cycle arrest observed.

LSCs reside within the BM niche, which serves as a sanctuary to facilitate self-renewal, proliferation and to avoid drug-induced intervention [58, 59]. Within the BM niche, leukaemic cells interact with many cell types, with the MSC stroma compartment important in protecting leukaemic cells from treatment. High levels of BMP’s secreted by the MSCs and CML cells is a key mechanism involved in providing protection from TKI’s. However, many in vitro co-culture studies are performed in 2D, which cannot recapitulate the cell–cell and cell–matrix interaction observed in the BM to secure LSC survival. To further investigate the effect of our inhibitors in stroma-leukaemic co-cultures we formed and cultured primary MSC spheroids with K562 cells in either microfluidic devices or in micropatterned plates. Our results confirmed that MSC spheroids are not affected by treatment but leukaemic cells in co-culture are, inducing apoptosis. Thus, all future co-culture studies should be carried out using 3D cell culture techniques to mimic the cellular characteristic in a more realistic way.

## Conclusion

Our results are a significant step towards overcoming the protective mechanisms/signals provided by the BM stroma which circumvent BCR::ABL1 directed therapy leading to LSC persistence. Here we demonstrate a therapeutic window whereby targeting the BMP pathway together with BCR::ABL1 and SFKs in CML is more effective than targeting BCR::ABL1 alone, both in 2D and 3D culture. New BMP receptor inhibitors are under development, particularly for application in other diseases such diffuse intrinsic pontine glioma (DIPG) [60, 61], and need to be assessed in the future work to enable single BMP-receptor inhibition in comparison to a simultaneous inhibition across different BMP receptors. Our observations emphasize the need of new combinatorial therapies targeting additional BCR::ABL1 independent pathways that promote the re-entry of quiescent cells back into the cell cycle, their differentiation through self-renewal pathway inhibition and their final elimination through TKIs. Therapeutic approaches which overcome BCR::ABL1 dependent and independent survival mechanisms by multi-kinase targeting using TKI such as SC are key to this goal. This ultimately could overcome LSC persistence, thereby improving the number of patients who achieve TFR in the future.

### Supplementary Information

Additional file 1

Additional file 2

Additional file 3

## Data Availability

All data generated or analysed during this study are included in this published article [and its supplementary information files].

## Abbreviations

ABL: Ableson leukaemia virus
BCR: Breakpoint cluster region
BFU-E: Burst-forming unit-erythroid
BM: Bone marrow
BMP: Bone morphogenetic protein
CFC: Colony forming cell
CFU-G: Colony-forming unit- granulocyte
CFU-GEMM: Colony-forming unit- granulocyte, erythroid, macrophage, megakaryocyte
CFU-GM: Colony-forming unit- granulocyte, macrophage
CFU-M: Colony-forming unit- macrophage
CI: Combination index
CML: Chronic myeloid leukaemia
CP: Chronic phase
CTV: Cell Trace Violet
DIPG: Diffuse intrinsic pontine glioma
DMEM: Dulbecco’s modified Eagle’s medium
dCt/ΔCt: Delta Ct
ddCt/ΔΔCt: Delta delta Ct
DOR: Dorsomorphin
ECM: Extracellular matrix
FDA: Fluorescein diacetate
FC: Fold change
GSEA: Gene set enrichment analysis
HSC: Haematopoietic stem cells
HSPC: Hematopoietic Stem and Progenitor Cell
IC50: Half maximal inhibitory concentration
IM: Imatinib
LSC: Leukaemic stem cells
MSC: Mesenchymal Stem Cell
PI: Propidium iodide
RTK: Receptor tyrosine kinase
SC: Saracatinib
SD: Standard deviation
SFK: SRC family kinases
SFM: Serum free medium
TKI: Tyrosine kinase inhibitor
