# Supplementary material for: Overcoming BCR::ABL1 dependent and independent survival mechanisms in chronic myeloid leukaemia using a multi-kinase targeting approach
Source: Cell Commun Signal. 2023 Nov 29;21:342. doi: 10.1186/s12964-023-01363-2 (PMC10685629; doi:10.1186/s12964-023-01363-2)
Supplement: Supplementary file 2 — Additional file 1 [file 12964_2023_1363_MOESM1_ESM.pdf]

Figure S1
Related to Figure 2.

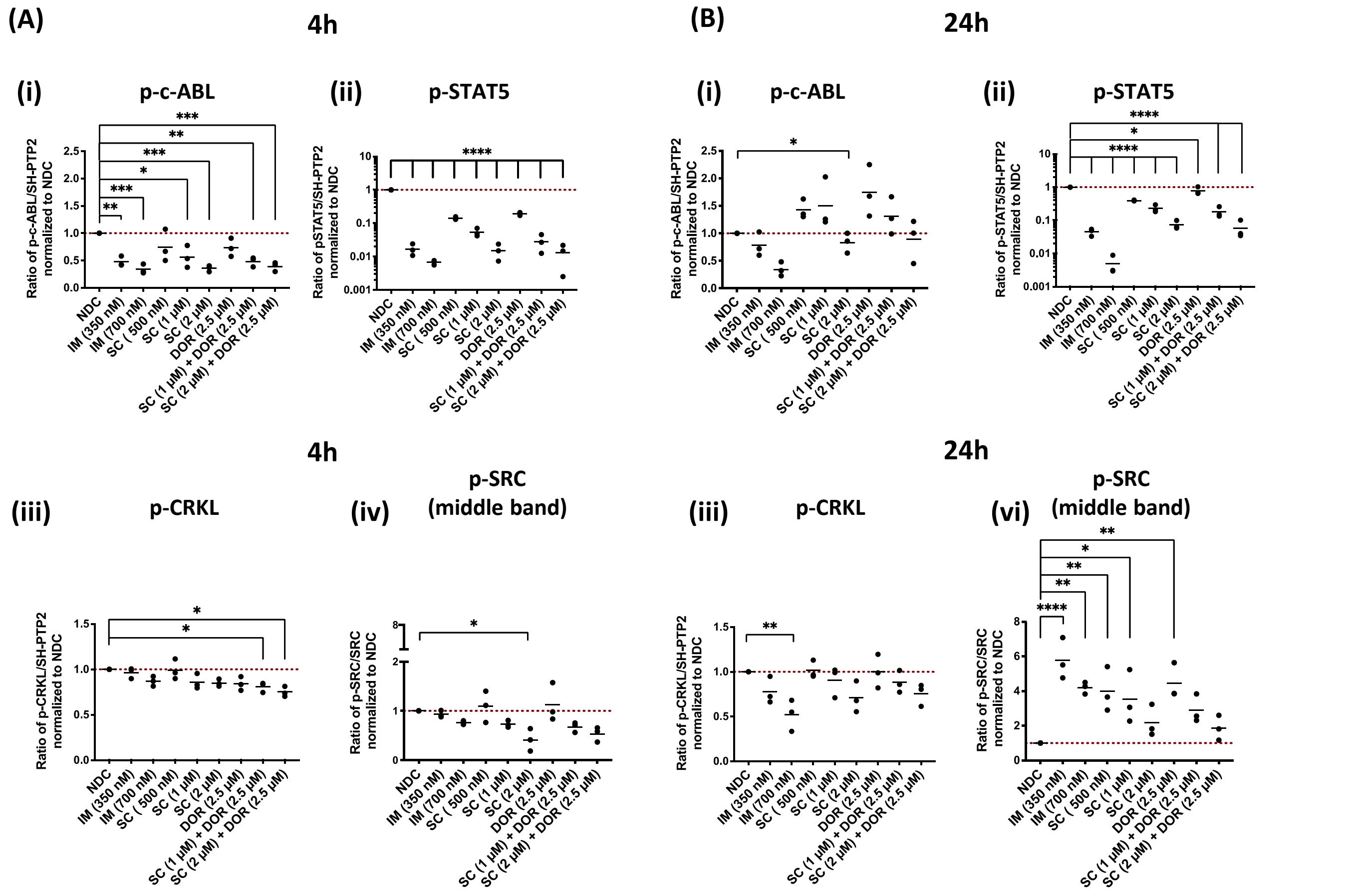

(C)

DOR [µM]	SC [µM]	Fa	CI
0.3125	0.25	0.46439	0.54426
0.3125	0.5	0.53548	0.422
0.3125	1	0.59952	0.3539
0.3125	2	0.60657	0.52596
0.625	0.25	0.48397	0.74705
0.625	0.5	0.59022	0.42029
0.625	1	0.66533	0.28904
0.625	2	0.62372	0.56744
1.25	0.25	0.57558	0.73046
1.25	0.5	0.57941	0.77875
1.25	1	0.63434	0.60646
1.25	2	0.61898	0.84819
2.5	0.25	0.64315	0.91314
2.5	0.5	0.65264	0.88728
2.5	1	0.68742	0.73731
2.5	2	0.68637	0.8181

(Ai-iv & Bi-iv) Quantification of immunoblot band intensities of the selected phosphorylated proteins over SH-PTP2 or total SRC (n=3). Protein expression of treated samples was compared to the NDC by One-Way-ANOVAs in GraphPad Prism 8. The grade of significance is indicated by asterisks (**** $p < 0.0001$, *** p 0.001 to 0.0001, ** p 0.01 to 0.001, * p 0.05 to 0.01). Bars represent mean ± SD. **(C)** The CI for all drug combinations is below 1, which defines synergy.

Figure S2
Related to Figure 3.

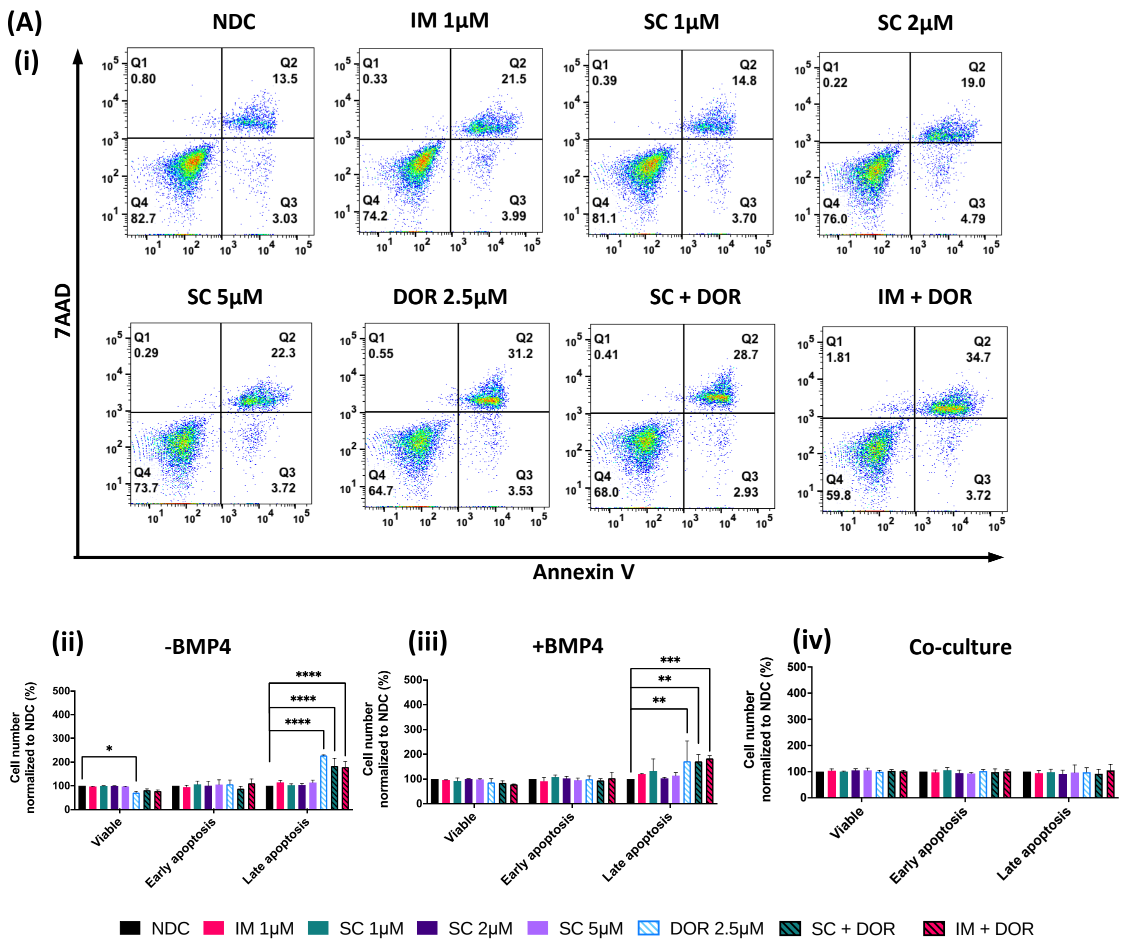

(Ai-ii) After 72 h treatment (IM= 1 µM, SC= 1 µM, 2 µM, 5 µM; DOR= 2.5µM; Combo= 1 µM TKI (IM or SC) + 2.5 µM DOR), apoptosis in normal $CD34^{+}$ cells indicate SC in combination with BMP pathway inhibitor DOR promotes modest, but significant increase, however to a smaller extent than in CP-CML samples. **(Aiii)** Inhibitor treatments with addition of BMP4 also indicate a significant increase of normal in late apoptosis. **(Aiv)** HS-5 co-cultures showed the same observed protective effect on treated normal $CD34^{+}$ cells as observed with CP-CML samples.

Figure S2
Related to Figure 3.

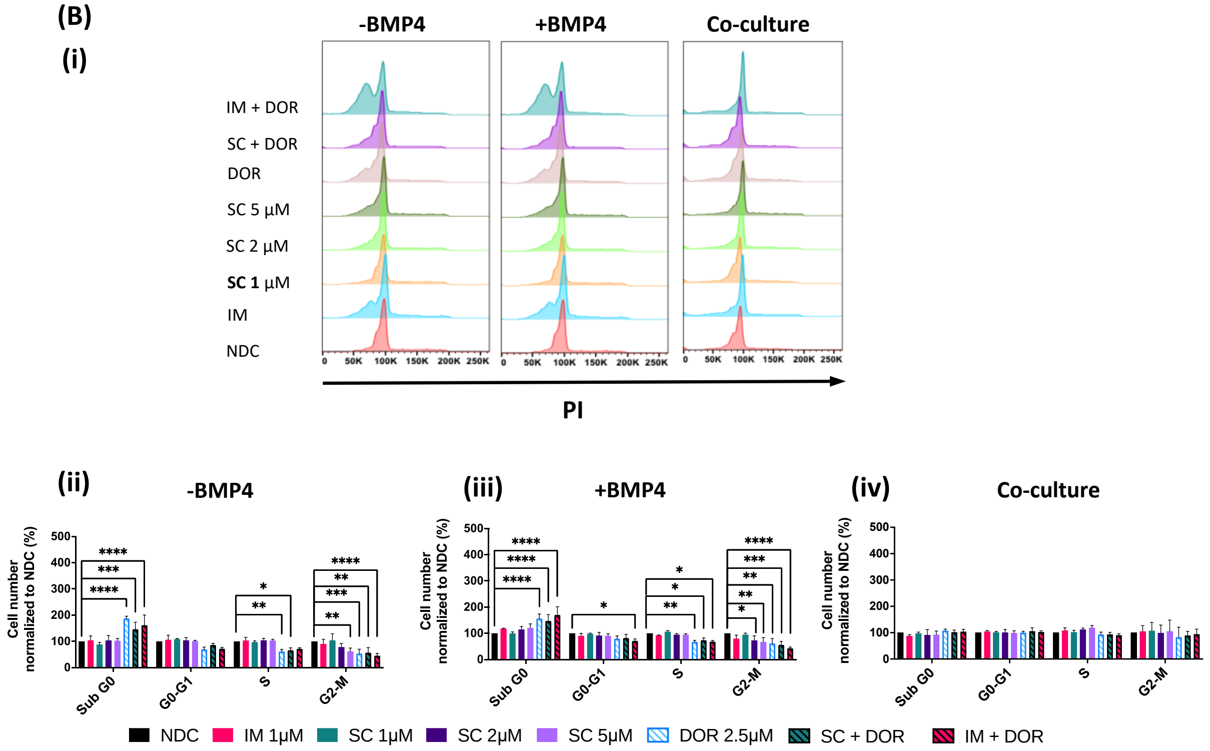

(Bi-iii) Cell cycle analyses revealed a significant increase in the number of cells in Sub-G0 in single and dual treatments with DOR independent of supplemented BMP4, but to a much lower extent compared to CP-CML samples. **(Biv)** Cell cycle analysis of co-cultures showed no changes upon treatment. Data are expressed as mean ± SD (n=3) and were compared using Two-Way-ANOVA (**** $p < 0.0001$, *** p 0.001 to 0.0001, ** p 0.01 to 0.001, * p 0.05 to 0.01).

Figure S3
Related to Figure 4.

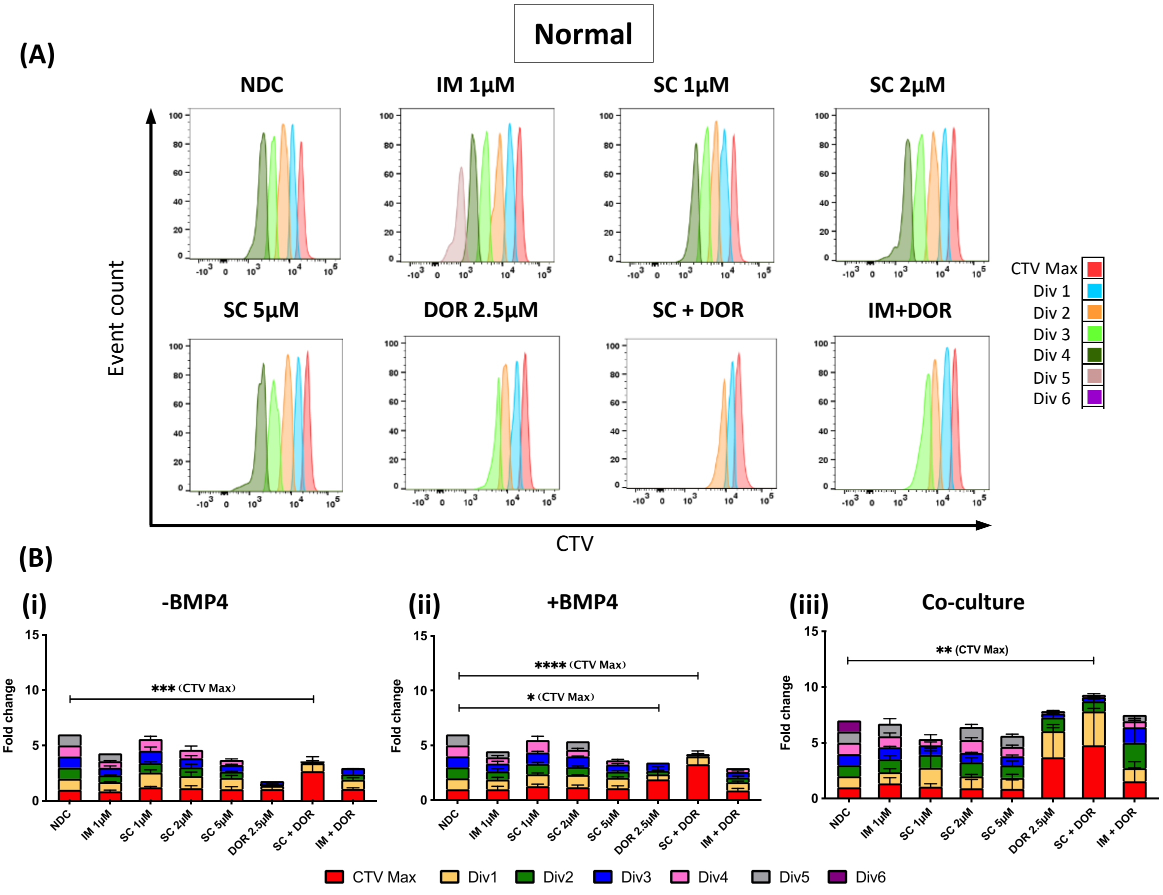

(**A & Bi-iii**) Proliferation analysis of normal $CD34^{+}$ samples treated with DOR alone or in combination with TKIs (IM= 1 µM, SC= 1 µM, 2 µM, 5 µM; DOR= 2.5µM; Combo= 1 µM TKI (IM or SC) + 2.5 µM DOR) caused cells to accumulate in early cell division, but to a smaller extent than CP-CML samples. Data are expressed as mean ± SD (n=3) and CTV_{max} and division 1 were compared using Two-Way-ANOVA (**** $p < 0.0001$, *** p 0.001 to 0.0001, ** p 0.01 to 0.001, * p 0.05 to 0.01).

Figure S4
Related to Figure 4.

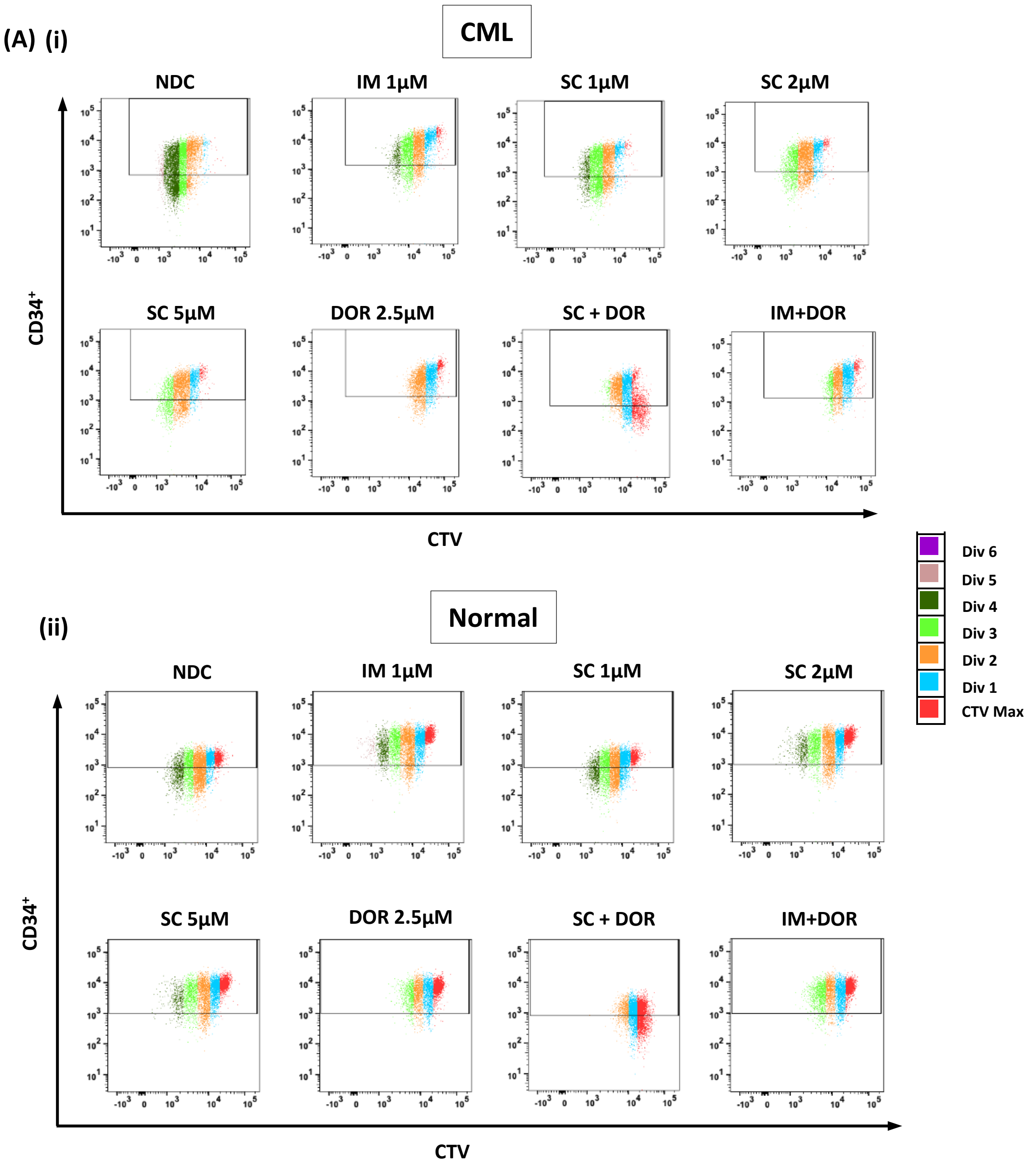

(A) Representative scatter plots overlay of one of the biological replicates of CP-CML (**Ai**) and normal **(Aii)** $CD34^+$ cells treated with inhibitors (IM= 1 µM, SC= 1 µM, 2 µM, 5 µM; DOR= 2.5µM; Combo= 1 µM TKI (IM or SC) + 2.5 µM DOR) in the absence of BMP4.

Figure S4
Related to Figure 4.

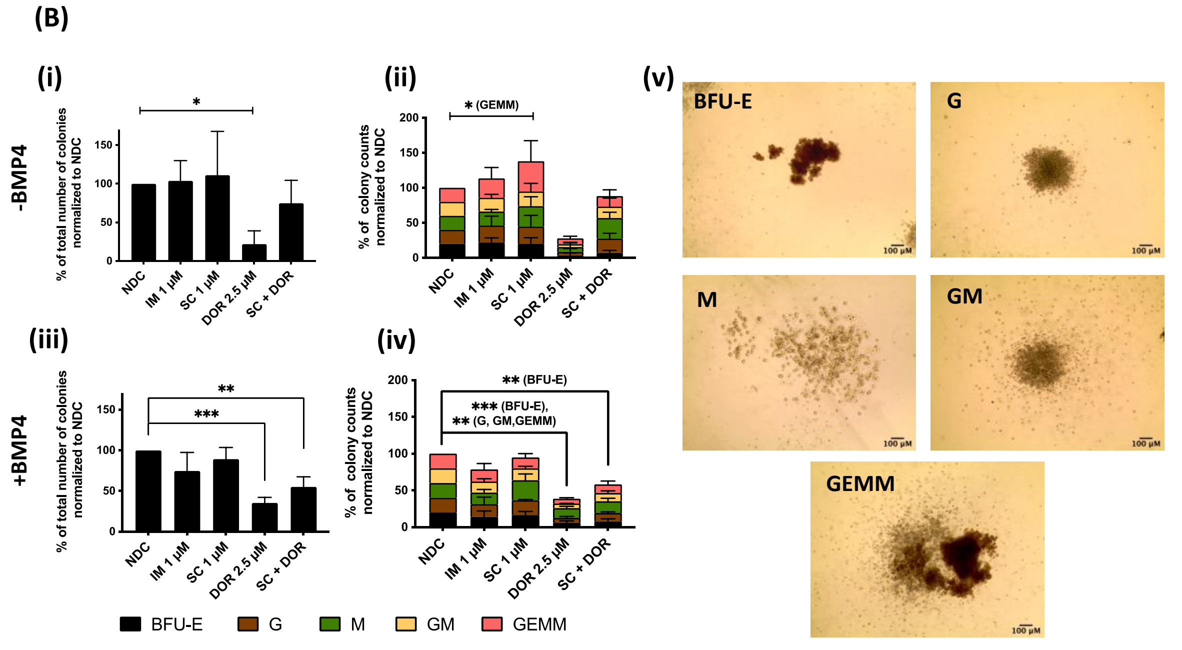

(Bi+iii) total colony counts and **(Bii+iv)** colony types independent of BMP4 supplementation. Results are displayed as mean ± SD (n=3) and normalized colony counts were compared to the NDC using Two-Way-ANOVA (**** p < 0.0001, *** p 0.001 to 0.0001, ** p 0.01 to 0.001, * p 0.05 to 0.01).

Figure S5
Related to Figure 5.

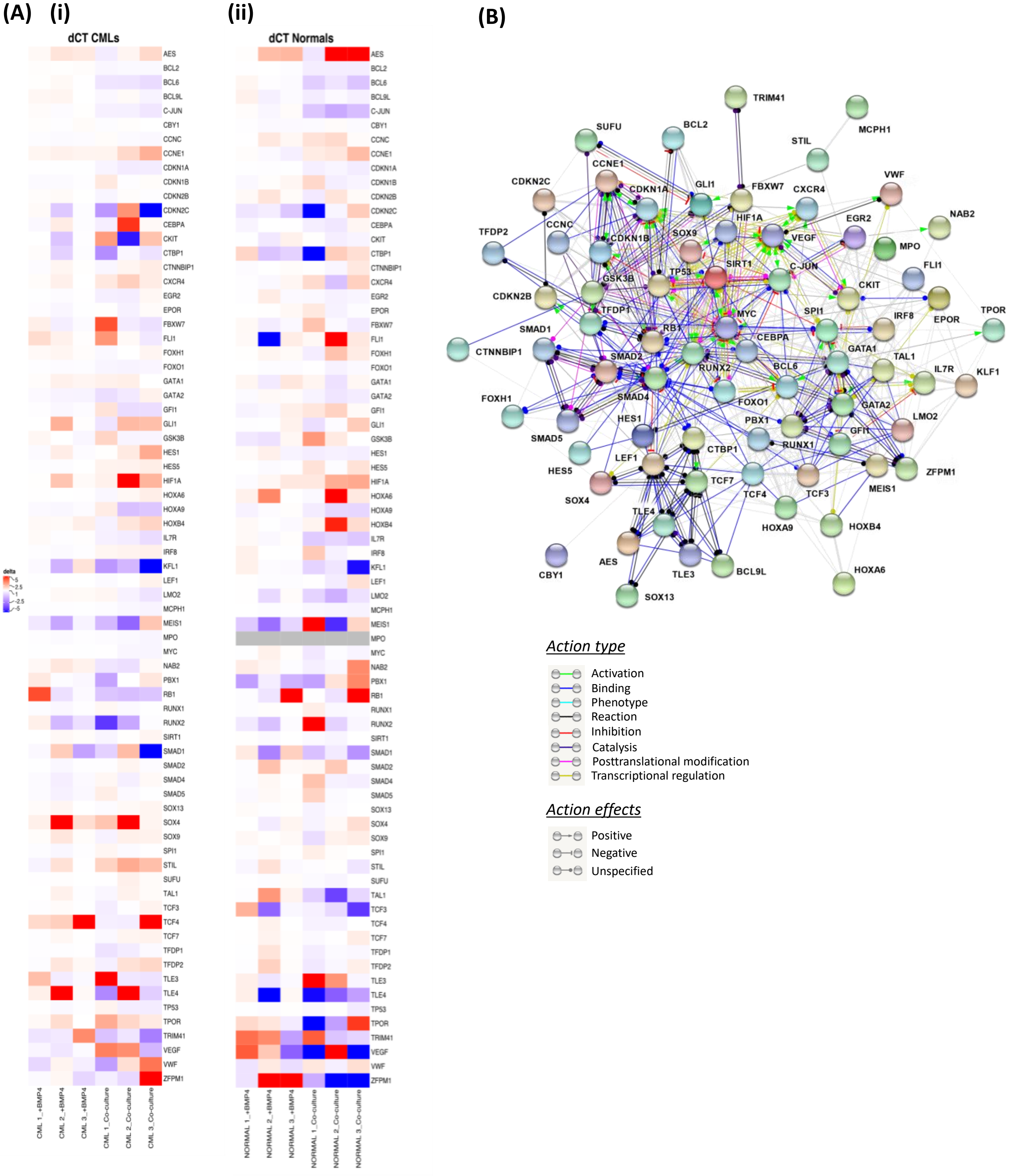

(Ai) CP-CML and **(Aii)** normal CD34^{+} cells were cultured with inhibitors (IM= 1 µM, SC= 1 µM, 2 µM, 5 µM; DOR= 2.5µM; Combo= 1 µM TKI (IM or SC) + 2.5 µM DOR) for 72 h in the absence or presence of BMP4 (20 ng/ml) or in stromal-co-culture with the cell line HS-5. Gene expression changes were analysed with dCt values normalized to NDC without BMP4 supplementation. Co-cultures resulted in a greater change in gene expression compared to monocultures supplemented with BMP4. (**B**) String network of chosen 73 genes, demonstrating the high level of interaction amongst all chosen targets.

Figure S6
Related to Figure 5.

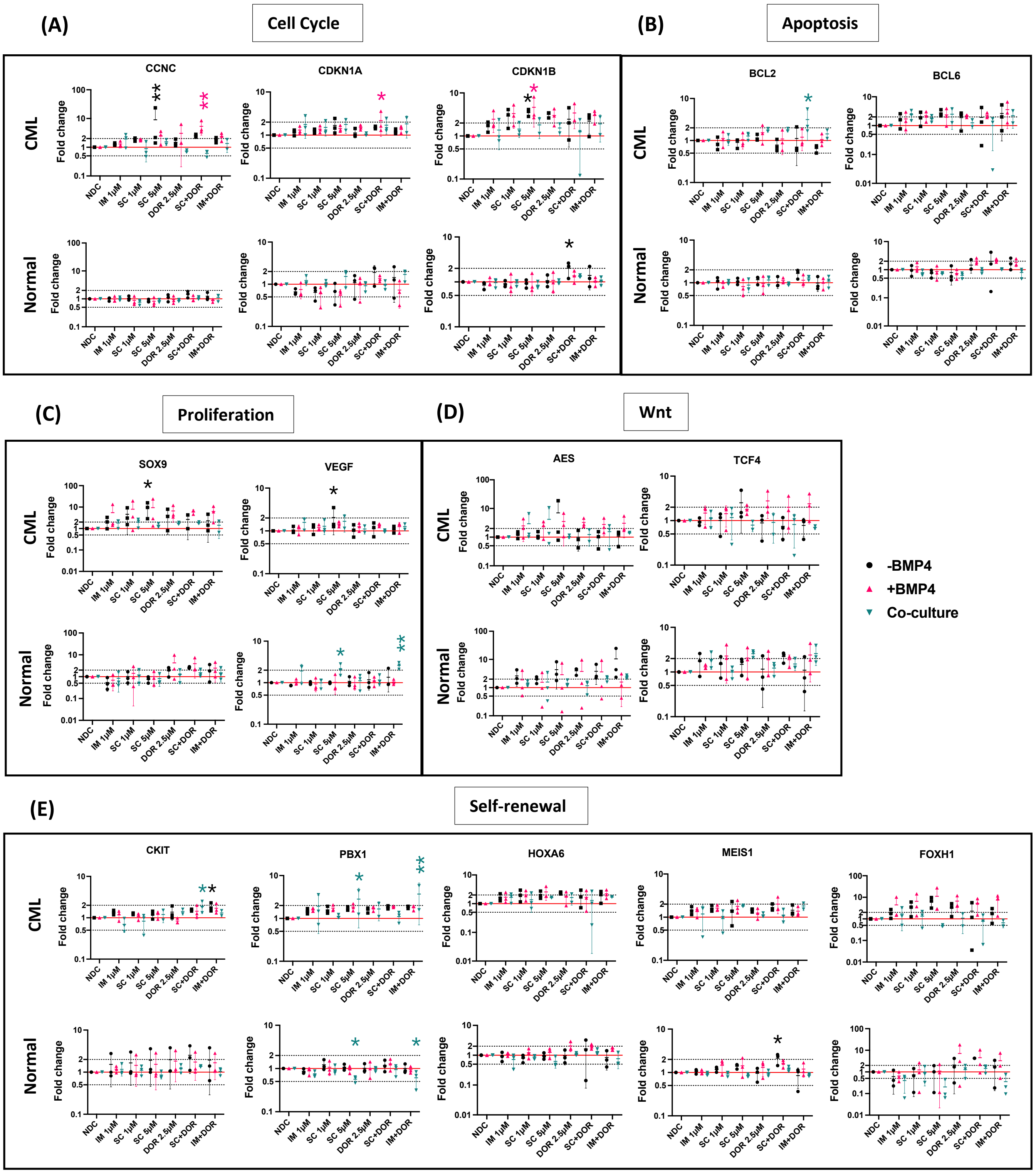

(A-E) Fluidigm gene expression analyses of CP-CML and normal CD34+ cells treated with the TKIs IM or SC and BMP receptor inhibitor DOR, and the combination (IM= 1 µM, SC= 1 µM, 2 µM, 5 µM; DOR= 2.5 µM; Combo= 1 µM TKI (IM or SC) + 2.5 µM DOR) in absence or presence of BMP4 (20 ng/ml), or in co-culture with HS-5 at 72 h. Selected genes can be grouped into (**A**) cell cycle, (**B**) apoptosis, (**C**) proliferation, (**D**) Wnt signalling and (**E**) self-renewal, and were identified to be deregulated after treatment. Selected genes most affected by treatments are express as fold change (2^{-ddCt}). Data are expressed as mean ± SD (n=3) and were compared using Two-Way-ANOVA (** p 0.01 to 0.001; * p 0.05 to 0.01).

Figure S7
Related to Figure 6.

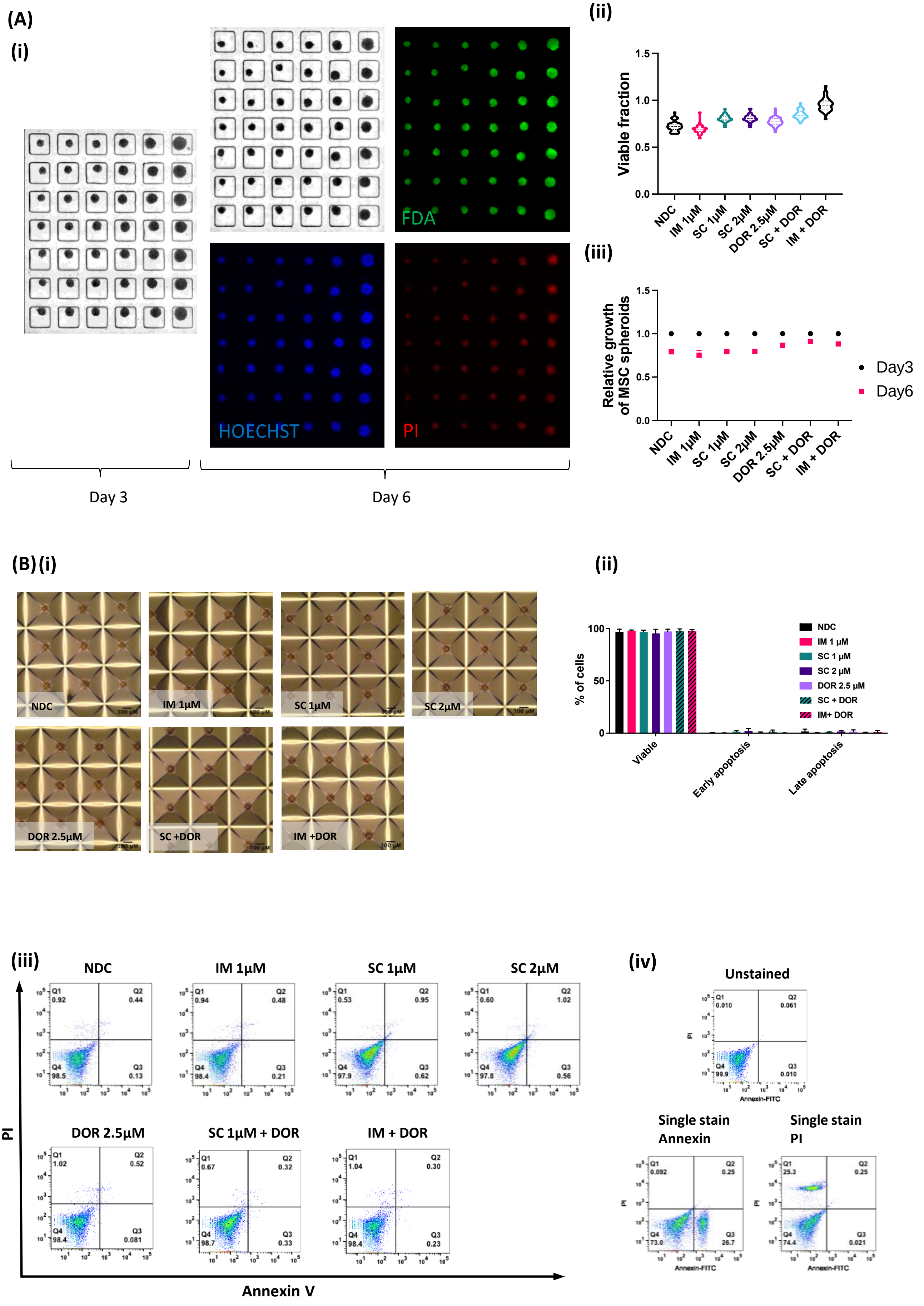

(Ai) MSC spheroids were formed in a microfluidic device and treated with DOR and TKIs (IM or SC) on day 3 post-seeding, for 72 h. Afterwards, spheroids were stained for viability using FDA, PI and Hoechst. **(Aii)** Viability and **(Aiii)** relative growth of spheroids was determined using MATLAB. Spheroid viability was not affected by inhibitor treatments and relative growth of spheroids was decreased on day 6 compared to day 3. The viable fraction was calculated based on the ratio of the FDA area on day 6 and the BF area on day 3 prior to treatment. The violin plot displays the median and no significant difference amongst treatments was found when performing One-Way-ANOVA. Green=FDA, Red=PI, Blue= Hoechst. **(Bi)** MSC spheroids in micropatterned plates were treated in a similar way to microfluidic devices. **(Bii-iv)** After 72 h treatment, cells were stained with Annexin V-FITC and PI to determine apoptosis. Apoptosis stains were measured by flow cytometry, expressed as mean ± SD (n=3) and were compared to the NDC by Two-Way-ANOVAs in GraphPad Prism 8.

Supplemental Material & Methods Related to Figure 6 and Figure S7.

Fluorescent images were processed in MATLAB software provided by the Zagnoni group to assess spheroid size before and after inhibitor treatment as well as the percentage of viable cells. To determine the relative spheroid growth the software detects the outline of each spheroid and calculates its volume knowing the dimension of the single microwell. Herby, we compared the same spheroid on day 3 and day 6 to calculate the relative growth; ***Area Day 6/ Area Day 3***. Fluorescent images were also analysed by calculating area sizes using MATLAB. Hereby, the area of BF images on day 3 (Area BF) when the drugs were added to the devices was divided by FDA positive area on day 6 (Area G); ***Area G/ Area BF***. For each treatment ~35 spheroids were analysed. Spheroids that were touching more than one side of the microwell had to be excluded to not calculate false area sizes. To study co-localization of leukaemic cells pre-labelled with CTV and live/dead stains, a co-localization plug-in called JACoP (Bolte and Cordelières, 2006) was run in ImageJ. For that, the background was subtracted but brightfield and contrast not further adjusted, as the plug-in only takes occurring signals into account but not intensities. Within the plug-in, Costes automatic threshold was applied. The resulting M2 coefficient (Live/Dead fraction overlapping with CTV fraction) for each PI and FDA stain were added up and set to 100 % to individually generate the percentage of live and dead stained cells, which are also CTV positive. Afterwards, all values were normalized to NDC.
