# Supplementary material for: Overcoming BCR::ABL1 dependent and independent survival mechanisms in chronic myeloid leukaemia using a multi-kinase targeting approach
Source: Cell Commun Signal. 2023 Nov 29;21:342. doi: 10.1186/s12964-023-01363-2 (PMC10685629; doi:10.1186/s12964-023-01363-2)
Supplement: Supplementary file 3 — Additional file 2 [file 12964_2023_1363_MOESM2_ESM.docx]

Supplemental Tables

Table 1

Oligonucleotide sequences of targeted qPCR

| **Target** | **Forward** | **Reverse** |
| --- | --- | --- |
| *AES* | CTGCCCTACCTCTCCCAAGA | TTGGAGCTGCTGTCGGATG |
| *BCL2* | CCCTGTGGATGACTGAGTACC | GTTCCACAAAGGCATCCCAGC |
| *BCL6* | GCACTTGGGACCACAGAGAAA | ATGCCTTGCTTCACAGTCCA |
| *BCL9L* | CGTACAGTGGGGACGAATGG | CATGGCTGGGTCTGCTACAT |
| *C-JUN* | CTTGAAAGCTCAGAACTCGGAG | CTGCGTTAGCATGAGTTGGC |
| *CBY1* | CGGAAGTCGGCATCTCTCTC | TTGCCCTGCCAGGTTCATAG |
| *CCNC* | CCCCTTGCATGGAGGATAGT | TCTGCTGTACAACACAGGCT |
| *CCNE1* | GACGGGGAGCTCAAAACTGA | CATGGCTTTCTTTGCTCGGG |
| *CDKN1A* | GTGGACCTGGAGACTCTCAG | CCTCTTGGAGAAGATCAGCCG |
| *CDKN1B* | GGCTAACTCTGAGGACACGC | TGAGTAGAAGAATCGTCGGTTGC |
| *CDKN2B* | GGTGGCTACGAATCTTCCG | CCTAAGTTGTGGGTTCACCA |
| *CDKN2C* | GGATTTGGAAGGACTGCGCT | TGACAGCGAAACCAGTTCGG |
| *CEBPA* | GCCAAGAAGTCGGTGGACAAG | TCATTGTCACTGGTCAGCTC |
| *CKIT* | GCATTCAAGCACAATGGCAC | CCAATCAGCAAAGGAGTGAA |
| *CTBP1* | CAGCTCGCACTTGCTCAAC | GTCTCGAGCCAAAGTGCTCA |
| *CTNNBIP1* | GGGCGGCACCTTCCT | GTTCATCCCCCTGCCTGG |
| *CXCR4* | TTACCATGGAGGGGATCAGT | GTAGATGGTGGGCAGGAAGA |
| *EGFR2* | TTGACCAGATGAACGGAGTG | TGGTTTCTAGGTGCAGAGACG |
| *EPOR* | ATGAGCCATGGAAGCTGTGT | CGTCTAGGAGCACTACTTCA |
| *FBXW7* | CCTTCTCTGGAGAGAGAAATGC | CTGTCTGATGTATGCACTTTTCC |
| *FLI1* | GCTGTTGTCACACCTCAGTT | GAGGACTTTTGTTGAGGCCA |
| *FOXH1* | ACCTTTCCTCCAACCGATGC | TGCCCAACCTTGGATGCTC |
| *FOXO1* | CTACGAGTGGATGGTGAAGAGC | CCAGTTCCTTCATTCTGCACTCG |
| *GATA1* | TATTCCTCTCCCAAGCTTCG | CATCTTGTGATAGAGGCCGCA |
| *GATA2* | GCGCAGCAAGGCTCGTT | AGCATTGCACAGGTAGTGGC |
| *GFI1* | GCTCCCCAGGACCAGACTAT | CTTCGGTCAGCTGCGATT |
| *GLI1* | AGCCAAGCACCAGAATCGG | TCTTGACATGTTTTCGCAGCG |
| *GSK3B* | CGACTAACACCACTGGAAGCT | GGATGGTAGCCAGAGGTGGAT |
| *HES1* | GGAGAAAAATTCCTCGTCCC | CGCGAGCTATCTTTCTTCAG |
| *HES5* | CATCCTGGAGATGGCTGTC | GAGCGTCAGGAACTGCAC |
| *HIF1A* | TACTCATCCATGTGACCATGAGG | TAGTTCTTCCTCGGCTAGTTAGG |
| *HOXA6* | GCAAAGCACTCCATGACGAAGG | GCCCATGGCTCCCATACACAG |
| *HOX9A* | CCCCATCGATCCCAATAACCC | TCCCTGGTGAGGTACATGTTG |
| *HOXB4* | CTGGATGCGCAAAGTTCACGTG | CGTGTCAGGTAGCGGTTGTAGT |
| *IL7R* | CACGATGTAGCTTACCGCCA | ATACATTGCTGCCGGTTGGA |
| *IRF8* | CCAGGACTGATTTGGGAGAA | AGTGGCTGGTTCAGCTTTGT |
| *KLF1* | ATCAGTACACTCACCACCCT | AACTTTCCAGGTTCCGAGTC |
| *LEF1* | CACTGACAGTGACCTAATGC | CAACGACATTCGCTCTCATT |
| *LMO2* | GGAGAGACTATCTCAGGCTT | CAGAAATGCTTCTGACAGGC |
| *MCPH1* | TCGCAAGCACCGCGTAG | ACCACACTTCAACATAGGCCA |
| *MEIS1* | AAGCAGTTGGCACAAGACACGG | CTGCTCGGTTGGACTGGTCTAT |
| *C-MET* | ATTTTGCTTTGCCAGTGGTGG | GACATGCCACTGTAAAGTTCCT |
| *MPO* | GCATCATCGGTACCCAGTTC | GTGGTGATGCCTGTGTTGTC |
| *MYC* | GACTCTGAGGAGGAACAAGA | TTGGCAGCAGGATAGTCCTT |
| *NAB2* | ACATCCTGCAGCAGACACTG | CAGCCCTTCCTCGAACTC |
| *PBX1* | GGAGGATACAGTGATGGACTCG | GGAGGTATCAGAGTGAACACTGC |
| *RB1* | AGGACCTGCCTCTCGTCAG | TCTCCCAAGTTAACCAAGCTCT |
| *RUNX1* | CACCTACCACAGAGCCATCAA | CTCGGAAAAGGACAAGCTCC |
| *RUNX2* | AACAAGACCCTGCCCGTGG | CATTCAGCAGAGGCATTCCGG |
| *SIRT1* | GCCTTGTTCTTTCCGCAGC | ACTAGAGCTTGCATGTGAGGC |
| *SMAD1* | GCTGCTCTCCAATGTTAACCG | CACTAAGGCATTCGGCATACAC |
| *SMAD2* | CCACGGTAGAAATGACAAGAAGG | GATTACAATTGGGGCTCTGCAC |
| *SMAD4* | GGGTCAACTCTCCAATGTCCAC | GTCACTAAGGCACCTGACCC |
| *SMAD5* | TGGGTCAAGATAATTCCCAGCCT | GGCTCTTCATAGGCAACAGGC |
| *SOX13* | GGATAGTGCTGACCCCCAAG | TCCGACACTCCTGGTCTCTT |
| *SOX2* | GCCCTGCAGTACAACTCCAT | GACTTGACCACCGAACCCAT |
| *SOX4* | GTTTAAACCACTGGATCTATCTAAATGCC | CACATATGCTATCATCATGCCATAAGAC |
| *SOX9* | ACCCGGATTACAAGTACCAGC | CCTTGAAGATGGCGTTGGGG |
| *SPI1* | ACAGGCGTGCAAAATGGAAG | AATACTCGTGCGTTTGGCGT |
| *STIL* | GACACAGTGCAAGCTGGAAGAC | AGTCAGGCTCTTGATCCTCACC |
| *SUFU* | CTCCAGGTTACCGCTATCGTCA | TGCTCAGGGATGTTGGCAGAAG |
| *TAL1* | CTATGAGATGGAGATTACTGATGGTC | GTGTGGGGATCAGCTTGC |
| *TCF3* | TGACCTCCTGGACTTCAGC | ACCTGAACCTCCGAACTGC |
| *TCF4* | AATCAAAACAGCTCCTCCGATT | CCATCTTGCCTCTTGGCCG |
| *TCF7* | TCAACCAGATCCTGGGTCGC | CCTTTCCTTGCGGGCCAG |
| *TFDP1* | GCTCTGGAGCCATACGTGAC | GGTCACTGGCAGAGAACCTT |
| *TFDP2* | ATAACCATTTGGCTGCTGATTCG | TCATTGCCATTAGCACATTTAAAGC |
| *TLE3* | GTCTTGTGACAGGATCAAAGACG | CATTTCAATGTTCAAGCCATAGGA |
| *TLE4* | GAAAACCACCAGGAGTTGACC | GTCAGCTCTCCGTTCATTCC |
| *TP53* | TTCTTGCATTCTGGGACAGCC | GGGGGTGTGGAATCAACCC |
| *TPOR* | GTCAATGGCAGCAACAGGAC | CCTCTTCGCAGTTCTCCCAG |
| *TRIM41* | CGAGAATCCAGGAGCCACAA | CTCCAGGTGCTTCCTCAGTG |
| *VEGF* | AGAAGGAGGAGGGCAGAATCA | AGGGTACTCCTGGAAGATGTCC |
| *VWF* | CAACACCTGCATTTGCCGAA | GCACACTGGACAGTCTCAAT |
| *ZFPM1* | GAGACATGTCCAGGCGGAAA | CTTTTGCTCCATGTGGCTGG |
| **House–keeping** |  |  |
| *ATP53* | TCCATCCTGTCAGGGACTATG | ATCAAACTGGACGTCCACCAC |
| *TYW1* | ATTGTCATCAAGACGCAGGGC | GTTGCGAATCCCTTCGCTGTT |
| *RNF20* | GGTGTCTCTTCAACGGAGGAA | TAGTGAGGCATCATCAGTGGC |
| *ENOX2* | GAGCTGGAGGGAACCTGATTT | CACTGGCACTACCAAACTGCA |
| *UBE2D2* | CCATGGCTCTGAAGAGAATCC | GATAGGGACTGTCATTTGGCC |

Table 2 Primary and secondary antibodies used for immunoblotting

| Antibody | Cat#/Manufacturer | Concentration |
| --- | --- | --- |
| p-c-Abl | #2861S/ Cell Signalling | 1:2000 |
| p-STAT5 | #9359S/ Cell Signalling | 1:2000 |
| p-Crkl | #3181S/ Cell Signalling | 1:2000 |
| Anti-Phosphotyrosine (4G10) | #05-321/ Millipore | 1:10,000 |
| p-Src | #2101/ Cell Signalling | 1:1000 |
| Src | #2110/ Cell Signalling | 1:1000 |
| SH-PTP2 | SC-7384/Santa Cruz | 1:2000 |
| GAPDH | #5174P/ Cell Signalling | 1:10,000 |
| IRDye 680RD donkey anti-mouse | LICOR | 1:5000 |
| IRDye 800CW donkey anti-rabbit | LICOR | 1:5000 |
